# Computational Modeling Analysis of Generation of Reactive Oxygen Species by Mitochondrial Assembled and Disintegrated Complex II

**DOI:** 10.3389/fphys.2020.557721

**Published:** 2020-10-16

**Authors:** Nikolay I. Markevich, Lubov N. Markevich, Jan B. Hoek

**Affiliations:** ^1^Institute of Theoretical and Experimental Biophysics RAS, Pushchino, Russia; ^2^Institute of Cell Biophysics of RAS, Pushchino, Russia; ^3^MitoCare Center for Mitochondrial Research, Department of Pathology, Anatomy, and Cell Biology, Thomas Jefferson University, Philadelphia, PA, United States

**Keywords:** complex II, reactive oxygen species, computational model, assembled, disintegrated

## Abstract

Reactive oxygen species (ROS) function as critical mediators in a broad range of cellular signaling processes. The mitochondrial electron transport chain is one of the major contributors to ROS formation in most cells. Increasing evidence indicates that the respiratory Complex II (CII) can be the predominant ROS generator under certain conditions. A computational, mechanistic model of electron transfer and ROS formation in CII was developed in the present study to facilitate quantitative analysis of mitochondrial ROS production. The model was calibrated by fitting the computer simulated results to experimental data obtained on submitochondrial particles (SMP) prepared from bovine and rat heart mitochondria upon inhibition of the ubiquinone (Q)-binding site by atpenin A5 (AA5) and Complex III by myxothiazol, respectively. The model predicts that only reduced flavin adenine dinucleotide (FADH_2_) in the unoccupied dicarboxylate state and flavin semiquinone radical (FADH^•^) feature the experimentally observed bell-shaped dependence of the rate of ROS production on the succinate concentration upon inhibition of respiratory Complex III (CIII) or Q-binding site of CII, i.e., suppression of succinate-Q reductase (SQR) activity. The other redox centers of CII such as Fe-S clusters and Q-binding site have a hyperbolic dependence of ROS formation on the succinate concentration with very small maximal rate under any condition and cannot be considered as substantial ROS generators in CII. Computer simulation results show that CII disintegration (which results in dissociation of the hydrophilic SDHA/SDHB subunits from the inner membrane to the mitochondrial matrix) causes crucial changes in the kinetics of ROS production by CII that are qualitatively and quantitatively close to changes in the kinetics of ROS production by assembled CII upon inhibition of CIII or Q-binding site of CII. Thus, the main conclusions from the present computational modeling study are the following: (i) the impairment of the SQR activity of CII resulting from inhibition of CIII or Q-binding site of CII and (ii) CII disintegration causes a transition in the succinate-dependence of ROS production from a small-amplitude sigmoid (hyperbolic) shape, determined by Q-binding site or [3Fe-4S] cluster to a high-amplitude bell-shaped kinetics with a shift to small subsaturated concentrations of succinate, determined by the flavin site.

## Introduction

Increasing interest in mitochondrial reactive oxygen species (ROS) production is caused by their crucial role not only in oxidative cellular damage and a development of various pathologies and aging but also in cell signaling that promote health by preventing a number of chronic diseases and extend lifespan (Ristow and Schmeisser, 2014). It was believed for a long time that complex I (CI) and respiratory Complex III (CIII) were the main producers of ROS in the respiratory chain (Turrens, 2003). The ability of respiratory complex II (CII) to generate ROS was debated, although there were experimental data (McLennan and Degli Esposti, 2000) allowing to conclude that CII can be a substantial source of ROS in mammalian mitochondria. It was found (McLennan and Degli Esposti, 2000) that CII is the predominant generator of ROS during prolonged respiration under uncoupled conditions, and CII appears to contribute to the basal production of ROS in cells.

The most clear evidence of CII of mammalian mitochondria to be a significant source of ROS under certain conditions was demonstrated in recent studies with inhibitors of CI, CII, and CIII (Quinlan et al., 2012; Siebels and Dröse, 2013; Grivennikova et al., 2017). It was found under condition when CI and CIII were inhibited in order to exclude ROS production from these complexes, CII could produce O_2_^−^/H_2_O_2_ in significant amounts, comparable and even exceeding ROS generated by CI and CIII, but only in the subsaturating range of succinate concentration. A dependence of the rate of ROS production on succinate concentration is bell-shaped with a maximum near 1,000 pmol/min mg prot at succinate concentration from approximately 50–500 μM in the experiments with both submitochondrial particles (SMP) and intact mitochondria (Quinlan et al., 2012; Siebels and Dröse, 2013; Grivennikova et al., 2017).

It was shown on SMP from bovine heart mitochondria (Siebels and Dröse, 2013) that excessive ROS production by CII at subsaturating succinate concentrations occurs due to a suppression of succinate-Q reductase (SQR) activity by different inhibitors such as atpenin A5 (AA5), blocker of ubiquinone (Q)-binding site, or stigmatellin, inhibitor of CIII. It was pointed out (Siebels and Dröse, 2013) that both inhibitors block SQR activity of CII: AA5 directly by blocking Q-binding site while the CIII inhibitor stigmatellin indirectly by decreasing the concentration of oxidized Q needed for the SQR activity. The suppression of SQR activity results in a strong succinate-dependent reduction of flavin adenine dinucleotide (FAD) and Fe-S redox centers located upper Q-binding site in CII that can induce significant ROS production by these centers at low succinate concentration.

While some authors (Quinlan et al., 2012) insist that only reduced FADH_2_ in the unoccupied dicarboxylate binding site, and probably, flavin semiquinone radical (FADH^•^) should be a generator of ROS to account for their experimental data, others (Grivennikova et al., 2017) believe that only terminal [3Fe-4S] cluster is most suitable for the role of a redox center that forms O_2_^−^, judging by the experimentally observed midpoint redox potential of a hypothetical electron donor for O_2_^−^of about 40 mV.

It was found (Lemarie et al., 2011) that a direct impairment of the SQR activity of CII that induces very high ROS production can occur as a result of CII disintegration. CII is composed of hydrophilic FAD‐ and iron-sulfur-containing subunits (SDHA and SDHB, respectively) bound to a two-subunit (SDHC and SDHD) hydrophobic membrane anchor that contains cyt *b* and Q-binding site. It was shown (Lemarie et al., 2011) that the SQR activity of CII can be specifically impaired without affecting the succinate dehydrogenase (SDH) activity of this CII. This is achieved by the specific dissociation of the SDHA/SDHB subunits, which encompass the SDH activity, from the membrane-bound SDHC/SDHD complex that is required for the SQR activity. Such disintegration of CII can result from the pH decline or mitochondrial Ca^2+^ influx (Hwang et al., 2014), and depends on the diphosphatidylglycerol cardiolipin (Schwall et al., 2012; Hwang et al., 2014). Besides, a broad range of human diseases from cancers to neurodegeneration related to SDH malfunction have recently been linked to defective assembly factors (Moosavi et al., 2019).

It was found recently (Korge et al., 2017) that the excessive ROS production by CII with bell-shaped dependence on succinate concentration under suppression of the SQR activity of CII can result in autocatalytic mitochondrial permeability transition (MPT) due to efflux of succinate from mitochondria through the open MPT pore and activate ROS production at low succinate concentration with following activation of apoptosis or necrosis/necroptosis.

Despite of the important role of CII as a ROS generator and a sensor of apoptosis, mechanisms of ROS formation by this complex remain insufficiently understood. First of all, there is no consensus as to which of the redox centers of CII really form O_2_^−^ and H_2_O_2_ with the bell-shaped dependence of the rates of ROS production on the succinate concentration observed experimentally (Quinlan et al., 2012; Siebels and Dröse, 2013; Grivennikova et al., 2017). Besides, it is not clear what changes occur in the kinetics of ROS formation by different sites of CII at CII transition from the assembled to disintegrated state. In order to answer some of these questions, a computational mechanistic model of electron transfer and O_2_^−^/H_2_O_2_ formation at different sites of CII in the assembled and disintegrated states is developed in the present study which is a continuation of our previous theoretical studies of CII (Markevich et al., 2019). Previously, we studied a simplified model of electron transfer in the assembled state of CII only and without heme *b* as an electron carrier in order to account for qualitatively experimentally observed high-amplitude bell-shaped responses of ROS production in CII upon inhibition of CIII. In the present study, the model is significantly extended by including heme *b* and consideration of CII in both assembled and disintegrated states. In addition, the model has been calibrated by fitting the computer simulated results to experimental data obtained on SMP prepared from bovine heart mitochondria upon inhibition of Q-binding site by AA5 (Siebels and Dröse, 2013) and from rat heart mitochondria upon inhibition of Complex III by myxothiazol (Grivennikova et al., 2017).

Kinetics of ROS generation by each redox center able potentially to form O_2_^−^or H_2_O_2_ was analyzed using the developed model under different conditions (assembled and disintegrated states of CII, inhibition of CIII, and Q-binding site of CII) to account for available experimental data on ROS production and make predictions to be tested experimentally.

## Methods and Models

### Kinetic Models of Electron Transfer in Assembled and Disintegrated CII

**Figure 1:**
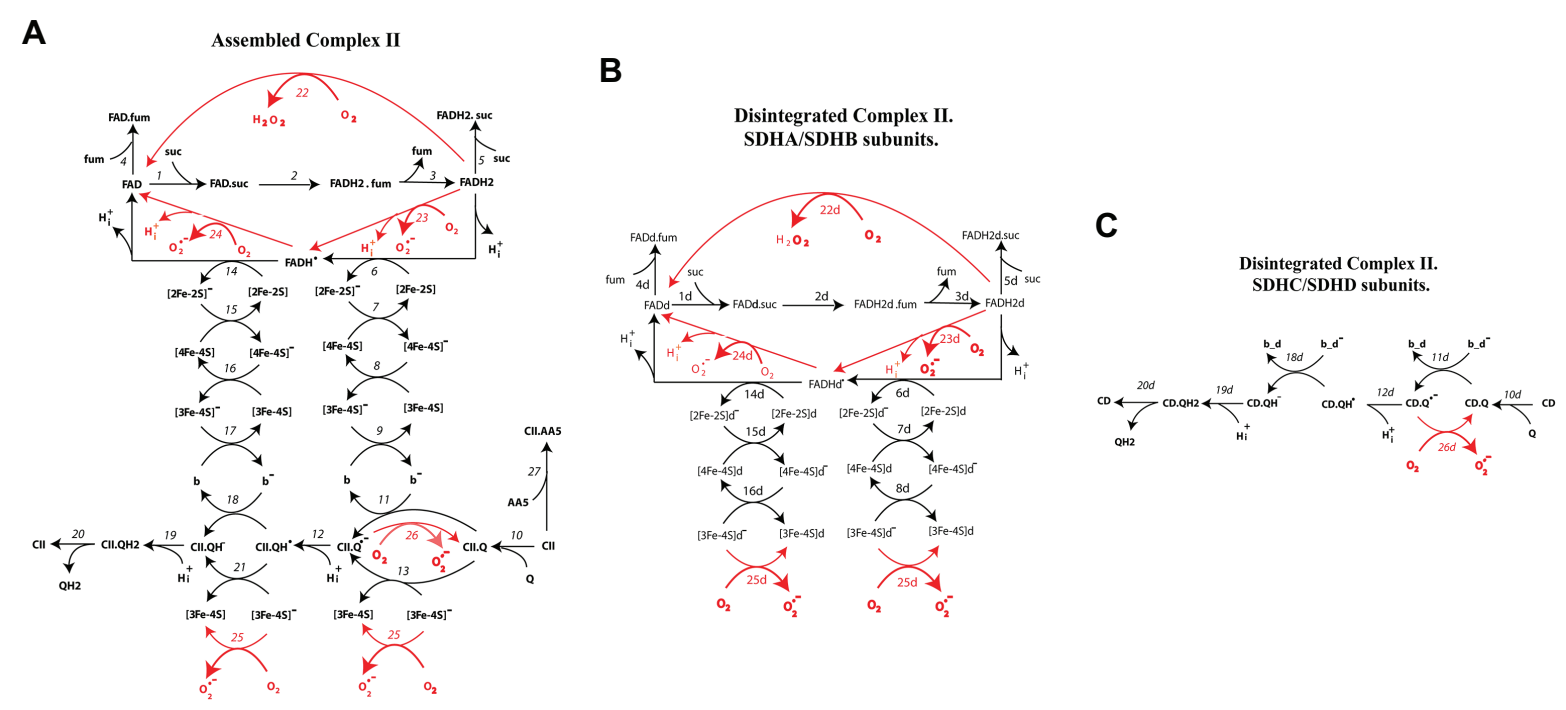
Kinetic schemes of electron transfer and formation of superoxide anion, O_2_^−^, and hydrogen peroxide, H_2_O_2_, in assembled **(A)** and disintegrated **(B,C)** Complex II. Reactions of O_2_^−^ and H_2_O_2_ formation are shown by red arrows. The detailed reaction network is presented in Supplementary Tables S1, S2.

Kinetic schemes of electron transfer and O_2_^−^/H_2_O_2_ production underlying a mechanistic computational model of CII in assembled and disintegrated states are presented in Figure 1. Figure 1A presents assembled CII, and Figures 1B,C – SDHA/SDHB and SDHC/SDHD subunits of disintegrated CII, respectively. Index “*d*” next to the names of redox centers and number reactions in Figures 1B,C means “disintegrated.” These kinetic schemes include the following electron carriers: (a) FADH_2_, (b) the sequence of iron-sulfur clusters: [2Fe-2S], [4Fe-4S], and [3Fe-4S], and (c) coenzyme Q. Electron transfer in CII takes into account both the mainstream electron pathway from succinate to Q (SQR activity) and bypass reactions resulting in O_2_^−^/H_2_O_2_ formation. These bypass reactions are marked in red in the kinetic schemes (Figure 1).

Electron pathway in kinetic schemes (A–C) can be described as follows.

**Figure 2:**
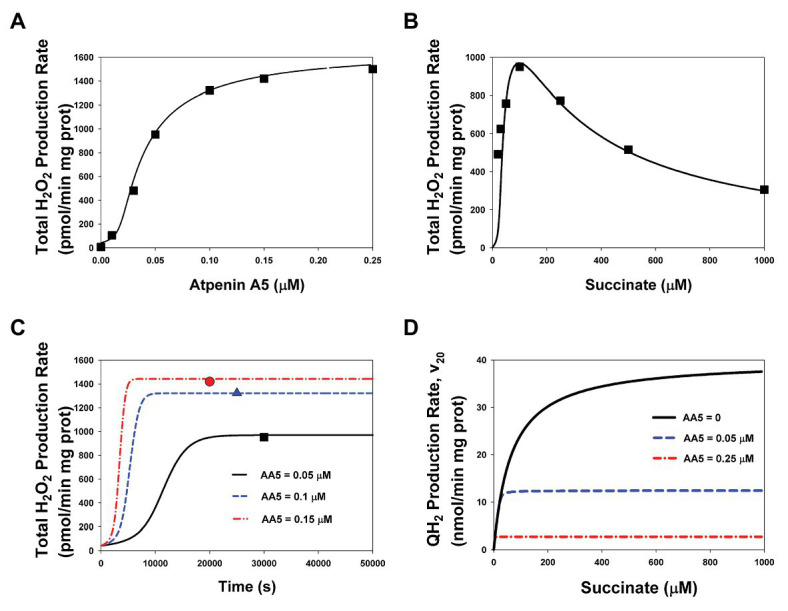
The stationary rates of total H_2_O_2_ and QH_2_ production by Complex II at varied concentrations of succinate and atpenin A5 (AA5). Computer simulation with the model of assembled respiratory Complex II (CII; lines) is compared to experimental data (symbols) on submitochondrial particles (SMP) prepared from bovine heart mitochondria (Siebels and Dröse, 2013). **(A,B)** The model simulated (solid line) and experimentally observed (black squares) dependence of the stationary rate of total H_2_O_2_ production on the AA5 concentration **(A)** (the concentration of succinate is equal to 100 μM) and the succinate concentration **(B)** (the AA5 concentration is equal to 0.05 μM). **(C)** Computer simulated time responses in the total rate of H_2_O_2_ production (lines) and its steady-state experimentally observed values [as shown in the section **(A)**] when the AA5 concentration changes from 0 to 0.05 (solid line and black squares), 0.1 (blue dashed line and triangles), and 0.15 (red dash-dot line and circles) μM. **(D)** The dependence of the computer simulated stationary rates of QH_2_ production (succinate oxidation) on the succinate concentration at varied AA5 concentration that are shown in the plane: AA5 = 0 (black solid line), AA5 = 0.05 (blue dashed line), and 0.25 (red dash-dot line) μM. All computer simulations were made at the total concentration of CII, CIIt, of 235 μM and k_29_ = 1 s^−1^. The rest model parameters are presented in Supplementary Tables S2, S3.

**Figure 3:**
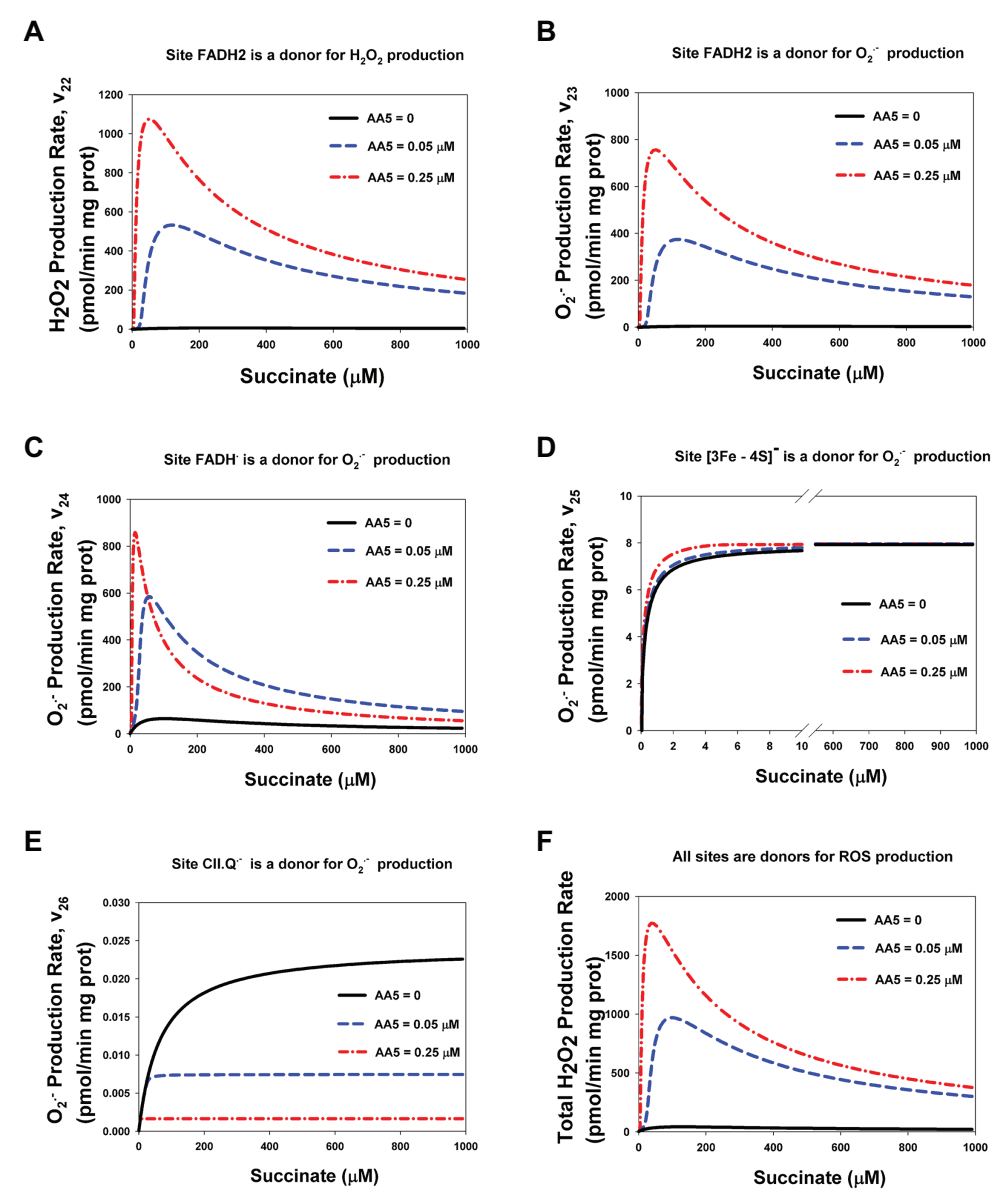
Computer simulation of the effect of AA5 on the stationary rate of reactive oxygen species (ROS) production by different redox centers of the assembled Complex II. **(A–F)** Model simulated dependencies of the stationary rates of H_2_O_2_ **(A,F)** and O_2_^−^ **(B–E)** production on the succinate concentration by different redox centers: flavin adenine dinucleotide (FADH_2_) **(A,B)**, FADH^•^ **(C)**, [3Fe-4S] cluster **(D)**, and CII-binding semiquinone CII.Q^−^ **(E)** at varied AA5 concentrations. **(F)** The total rate of H_2_O_2_ production by CII. Black solid, blue dashed, and red dash-dot lines correspond to AA5 = 0, 0.05, and 0.25 μM, respectively. All computer simulations were made at model parameter values as for Figure 2.

After reduction in the reactions (1–3) in Figure 1A and (1d–3d) in Figure 1B, FADH_2_ donates the first electron to the [2Fe-2S] cluster [reactions (6) in Figure 1A and (6d) in Figure 1B]. Then, the first electron through cluster [4F-4S] [reactions (7, 7d)] goes to the terminal cluster [3F-4S] [reactions (8, 8d) in Figures 1A,B, respectively]. In the assembled CII oxidized Q, binds to the Q-binding site [CII.Q site, reaction (10)], thus, the first electron can transfer from [3F-4S] cluster to Q-binding to CII either indirectly through cyt *b* [reactions (9, 11)] or directly in the reaction (13). Thus, transfer of the first electron from FADH_2_ to the CII.Q results in generation of semiquinone anion (CII.Q^−^) in the reactions (11, 13). Then, semiquinone anion binds proton H^+^ forming protonated semiquinone radical CII.QH in the reaction (12).

Potential sites of ROS generation in this branch of the first electron transfer in Figure 1A are FADH_2_, CII.Q^−^, and [3Fe-4S]. FADH_2_ can generate either H_2_O_2_ in the reaction (22) or superoxide in the reaction (23). Semiquinone, CII.Q, and [3Fe-4S] clusters generate superoxide in the reactions (25, 26). The same events occur in the matrix located subcomplex SDHA/B (Figure 1B) that is dissociated from the membrane-anchoring SDHC/D subcomplex. Difference between assembled and disintegrated subcomplex SDHA/B is only in downstream electron transfer. Oxidized [3Fe-4S]^−^ cluster in disintegrated CII cannot donate electron to the cyt *b* and CII binding Q located in the membrane. In this case, [3Fe-4S]^−^ donates electron to the oxygen only forming superoxide in the reaction (25d; Figure 1B).

By analogy, the second electron from the FADH˙ radical transfers to the protonated semiquinone CII.QH˙ in the reactions (14–18 and 21) with following formation and releases QH2 to the matrix in the reactions (18–21). In this branch of the second electron transfer, potential sites of superoxide formation are the FADH˙ radical [reaction (24)] and [3Fe-4S]^−^ [reaction (26)]. In addition, reaction (27) describes binding of AA5, inhibitor of CII, to the Q-binding site forming the inactive complex CII.AA5. In this reaction, AA5 competes with Q for binding to the Q-binding center, and thus competitively inhibits CII.

The entire reaction network of electron transfer and ROS production corresponding to kinetic schemes in Figure 1 consists of 53 reactions and is described in detail in Supplementary Table S1. The values of midpoint redox potentials, rate constants, and concentrations of different electron carriers were taken from experimental data and presented in Supplementary Table S2.

### Computational Model of Electron Transfer in Assembled and Disintegrated CII

A computational model consisting of 35 ordinary differential equations (ODEs) and 15 moiety conservation equations was derived from the reaction network using the law of mass action and Michaelis kinetics for all 53 kinetic processes. The model was implemented in DBSolve Optimum software available at http://insysbio.ru. The details of the mathematical model describing oxidized and reduced states of different carriers and electron flows through complex II are presented in Supplementary Data. The kinetic parameters used in the model are consistent with either measured or estimated values reported in the literature. We initially assumed the parameter values specified in the work (Markevich et al., 2019). They are presented in Supplementary Table S2. The simulated dependence of the rate of ROS production by CII on the succinate concentration demonstrated a good fit to the experimental data upon inhibition of Complex III. We subsequently fitted model simulation results to various experimental data, i.e., optimized some parameter values within experimental or estimated constraints to minimize the square deviation of the residuals, ∑(Res*_i_*)^2^, where each residual (Res*_i_*) is the difference between the experimental data point *i* and the value calculated by numerical computation of the model in the steady state for the given parameter set. Finding stationary solutions of the model, i.e., solving a system of algebraic equations, and the fitting procedure was performed by the DBSolve Optimum software in the Implicit Solver and Fitter options, respectively. Supplementary Table S3 lists the values of the adjustable kinetic parameters.

Additionally, the model is presented in SBML format by separate file: 2019_cII_Final.xml as supporting information.

## Results and Discussion

### Assembled CII

In order to investigate what redox centers of CII responsible for the experimentally observed high-amplitude bell-shaped dependence of the total rate of ROS generation by CII on the succinate concentration upon inhibition of CIII or Q-binding site (Quinlan et al., 2012; Siebels and Dröse, 2013; Grivennikova et al., 2017), i.e., suppression of the SQR activity of assembled CII as was pointed in Siebels and Dröse (2013), kinetics of ROS formation by each redox center able potentially to form O_2_^−^ or H_2_O_2_ was analyzed computationally using mathematical modeling simulation of different inhibitory conditions.

#### Computer Simulated Inhibition of the Q-Binding Site of Assembled CII

Initially, in order to calibrate the developed model, computer simulated dependencies of the rates of ROS production on the AA5, inhibitor of the Q-binding site, and succinate concentration were fitted to experimental data on SMP prepared from bovine heart mitochondria (Siebels and Dröse, 2013). Computer simulated results and experimental data presented in Figure 2 show a good fit for both dependencies of the H_2_O_2_ production rate on the AA5 concentration at the fixed succinate concentration (100 μM; Figure 2A) and on the varied succinate concentration at the fixed AA5 concentration (0.05 μM; Figure 2B). Fitting resulted in changes in values of some model parameters compare to the initial values including literature data. The new adjustable values of some parameters are presented in Supplementary Table S3. The most significant deviations of the adjusted values from the initial values are observed for the catalytic constants of the rate of ROS generation by each redox center and the catalytic constants of Q binding to the Q-binding center. Computer simulated time course of the total H_2_O_2_ production rate at changes in the AA5 concentration from 0 up to 0.05, 0.1, and 0.15 μM at fixed succinate concentration (100 μM; Figure 2C) confirms stationary fit computer simulated and experimental data presented in Figure 2A. Computer simulated dependencies of the stationary rate of QH2 production (succinate oxidation) on the succinate concentration at different fixed AA5 concentrations presented in Figure 2D show that the maximum reaction rate and the Michaelis constant decrease proportionally with increasing AA5 concentration which is typical for a competitive inhibitor.

Computer simulated dependence of stationary rates of ROS production by different redox centers of CII, namely: the flavin site, [3Fe-4S] cluster, and semiquinone, CII.Q^−^, at the Q-binding site on the succinate concentration with varying degrees of inhibition of the Q-binding site by AA5 are presented in Figure 3. These computer simulation results predict that only reduced FADH_2_ in the unoccupied dicarboxylate state (Figures 3A,B) and FADH^•^ (Figure 3C) have the experimentally observed [5] bell-shaped dependence of the rate of ROS production on the succinate concentration upon inhibition of the Q-binding site by AA5. Succinate-dependence of the rate of ROS formation at these sites (Figures 3A–C) as well as the total rate of ROS generation by CII (Figure 3F) changes from a very small amplitude in the basal state (AA5 = 0) to the intermediate‐ and high-amplitude bell-shaped kinetics with a shift to small succinate concentration upon an intermediate (AA5 = 0.05 μM) and strong (AA5 = 0.15 μM) inhibition of the Q-binding site, respectively.

It is proposed that FADH_2_ can generate both hydrogen peroxide, H_2_O_2_, and superoxide, O_2_^−^, with the rates v_22_ and v_23_, respectively (Figure 1A; Supplementary Table S1). The ratio of the catalytic constants of H_2_O_2_, and O_2_^−^ formation k_22_ and k_23_ are unknown, one authors (Siebels and Dröse, 2013) found 75% H_2_O_2_ and 25% O_2_^−^ in rat heart mitochondria while another (Grivennikova et al., 2017) found that bovine heart mitochondrial respiratory CII generates ROS, mostly as superoxide. Our fitting results predict close values for these constants: k_22_ = 0.027 μM^−1^⋅s^−1^ and k_23_ = 0.019 μM^−1^⋅s^−1^, so v_22_ (Figure 3A) and v_23_ (Figure 3B) look similar although we have to take into account that v_23_ is the rate of O_2_^−^ production, so the contribution of v_23_ in the total rate of H_2_O_2_ production two times less than v_22_ because two molecules O_2_^−^ give one molecule H_2_O_2_ in the process of subsequent dismutation (reaction 28) in Supplementary Table S1. The stationary total rate of H_2_O_2_ production by CII (vH_2_O_2tot_) was computed as the rate of H_2_O_2_ release from the mitochondrial matrix to cytosol that equal to the summary rate of H_2_O_2_ production by FADH_2_ in assembled, v_22_, and disintegrated, v_22d_, states and dismutation of O_2_^−^, v_28_, in the matrix at the steady state (see *Explicit functions* in Mathematical model in Supplementary Data).

The computer simulated dependence of stationary rates of ROS (O_2_^−^) production by [3Fe-4S] cluster, v_25_, (Figure 3D) and semiquinone at the Q-binding site, v_26_, (Figure 3E) on the succinate concentration show monotonic hyperbolic kinetics in the basal state (AA5 = 0) as well as upon an intermediate (AA5 = 0.05 μM) and strong (AA5 = 0.25 μM) inhibition of the Q-binding site by AA5. It should be pointed that AA5 increases the sensitivity of O_2_^−^ production by [3Fe-4S] cluster, v_25_, to succinate, i.e., decreases Michaelis constant, K_m25_, and does not affect on the maximal rate v_25_. On the contrary, AA5 decreases proportionally the maximal rate of O_2_^−^ production by semiquinone at the Q-binding site, v_26_, and K_m26_.

#### Computer Simulated Inhibition of CIII

**Figure 4:**
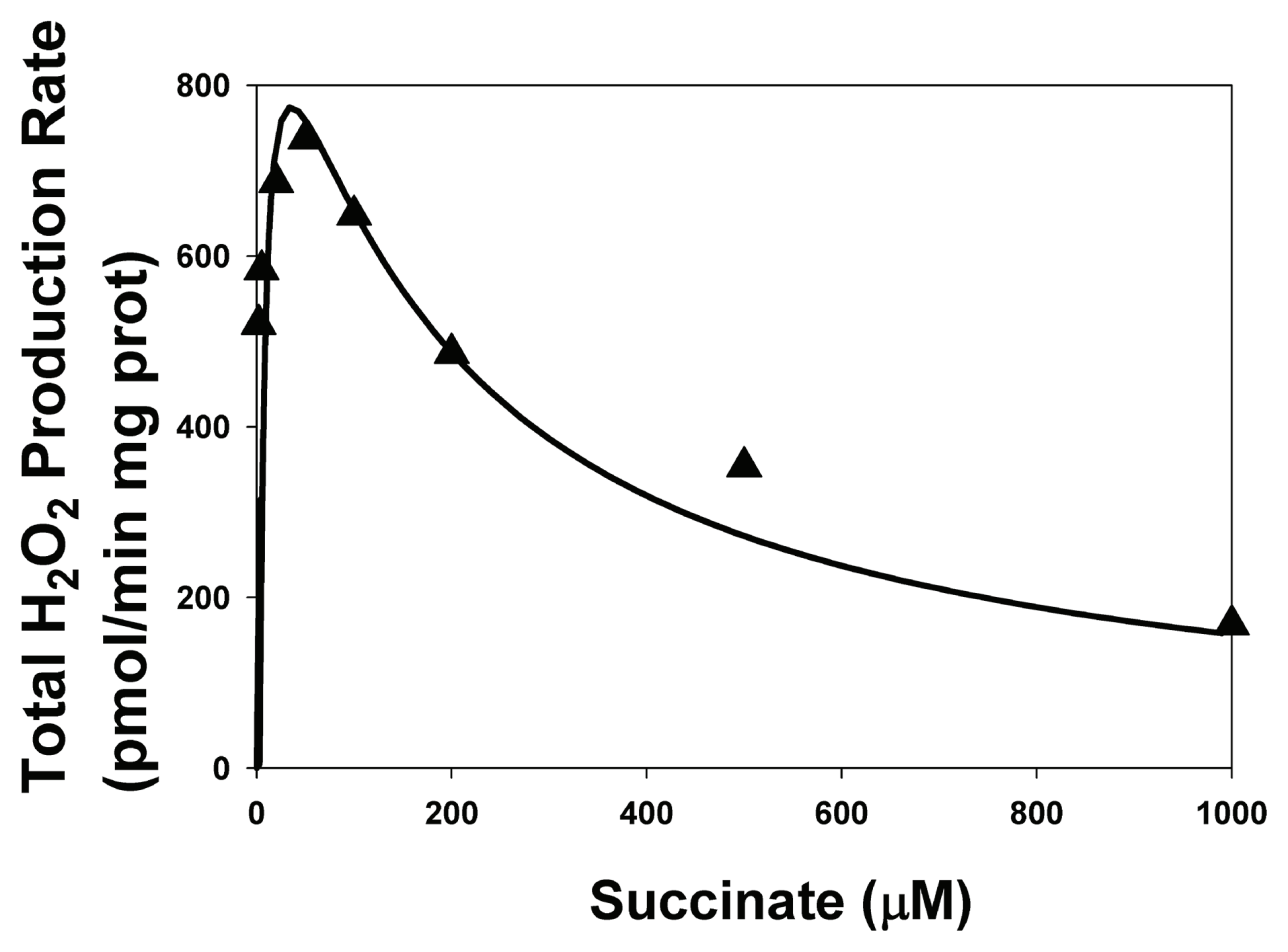
The dependence of the stationary rate of total H_2_O_2_ production by CII on the succinate concentration upon inhibition of Complex III. Computer simulation (line) is compared to experimental data (black triangles) on SMP from rat heart mitochondria upon inhibition of Complex III by myxothiazol (1.6 ìM; Grivennikova et al., 2017). All computer simulations were made at model parameter values as for Figure 2 except AA5 = 0, k_29_ = 0.005 s^−1^, and the total concentration of CII, CIIt, equal to 97 μM.

Just like in the work (Markevich et al., 2019), inhibition of CIII was simulated by decreasing the catalytic constant k_29_ of QH_2_ oxidation in the mitochondrial inner membrane [reaction (29) in Supplementary Tables S1, S2]. Figure 4 shows that model simulation of the stationary rate of H_2_O_2_ production at k_29_ = 0.005 s^−1^ (k_29_ = 1 s^−1^in the uninhibited state) good fit to experimental data on SMP from rat heart mitochondria upon inhibition of Complex III by myxothiazol (1.6 ìM; Grivennikova et al., 2017). It should be emphasized that values of all parameters including adjustable are the same for both modeling simulation presented in Figures 2A,B, 4 except the total concentration of CII, CIIt, that is equal to 235 and 97 μM for Figures 2, 4, respectively. This fact strongly suggests that the calibration of the developed mathematical model is correct.

Computer simulation modeling results upon inhibition of CIII confirm suggested earlier hypothesis that the non-monotonic succinate-dependence of the rate of ROS generation in CII results from ROS formation at the flavin in the unoccupied dicarboxylate binding site (Quinlan et al., 2012). Computational modeling analysis shows that only FADH_2_ and FADH^•^ have the experimentally observed bell-shaped dependence of the rate of ROS production on the succinate concentration upon inhibition of CIII and predict that inhibition of CIII has the same effects on the kinetics of ROS production by different CII redox centers (Supplementary Figure S1) as inhibition of the Q-binding site considered above (Figure 3). Such similar effect of inhibition of CIII and Q-binding site on the total ROS production by CII was pointed earlier as a result from suppression of SQR activity of CII (Siebels and Dröse, 2013). The stronger inhibition of CIII (a more decrease in k_29_) results in the more amplitude of the bell-shaped succinate-dependence of the rate of ROS production by the flavin site (Supplementary Figures S1A–C), and the total rate of ROS production by CII (Supplementary Figure S1F) and more shift of the maximal rate of ROS production to the small succinate concentration. The dependence of the stationary rate of ROS formation by [3Fe-4S] cluster, v_25_, (Supplementary Figure S1D) and semiquinone at the Q-binding site, v_26_, (Supplementary Figure S1E) on the succinate concentration shows hyperbolic kinetics in the basal state (k_29_ = 1 s^−1^) as well as upon an intermediate (k_29_ = 0.1 s^−1^) and strong (k_29_ = 0.01 s^−1^) inhibition of CIII with very small amplitude. Thus, these computer simulation data predict that the effect of inhibition of CIII and the Q-binding site on ROS production by different sites of CII is very similar.

#### Bell-Shaped vs. Hyperbolic Kinetics of ROS Production and SQR Activity. What the Reason?

The hypothesis that a decrease in the rate of ROS production by flavin in the unoccupied dicarboxylate binding site at the high succinate concentration may be a reason for bell-shaped dependence of the total rate of ROS production by CII upon inhibition of CIII was first proposed in (Quinlan et al., 2012). It is really easy to understand a decrease in the concentration of ROS-producing site unoccupied FADH_2_ due to succinate binding to FADH_2_ in the reaction (5; Figure 1A; Supplementary Table S1). More difficult to understand hyperbolic dependence of the rate of ROS formation on the succinate concentration by [3Fe-4S] cluster and semiquinone at the Q-binding site as well as the concentration of these sites and other Fe-S clusters (Supplementary Figure S2) located downstream flavin. Moreover, experimentally observed catalytic activity of CII has also hyperbolic dependence on the succinate concentration (Quinlan et al., 2012), although at the first glance, bypass ROS production rate by these sites as well as the mainstream electron flow in CII, i.e., the rate of QH_2_ production, v_20_, should have non-monotonic dependence on the succinate concentration because of bell-shaped dependence of the concentration of FADH_2_ and FADH^•^ (Supplementary Figure S2) that are substrate and product, respectively, in the mainstream and bypass reactions (6) and (23) (Figure 1A).

**Figure 5:**
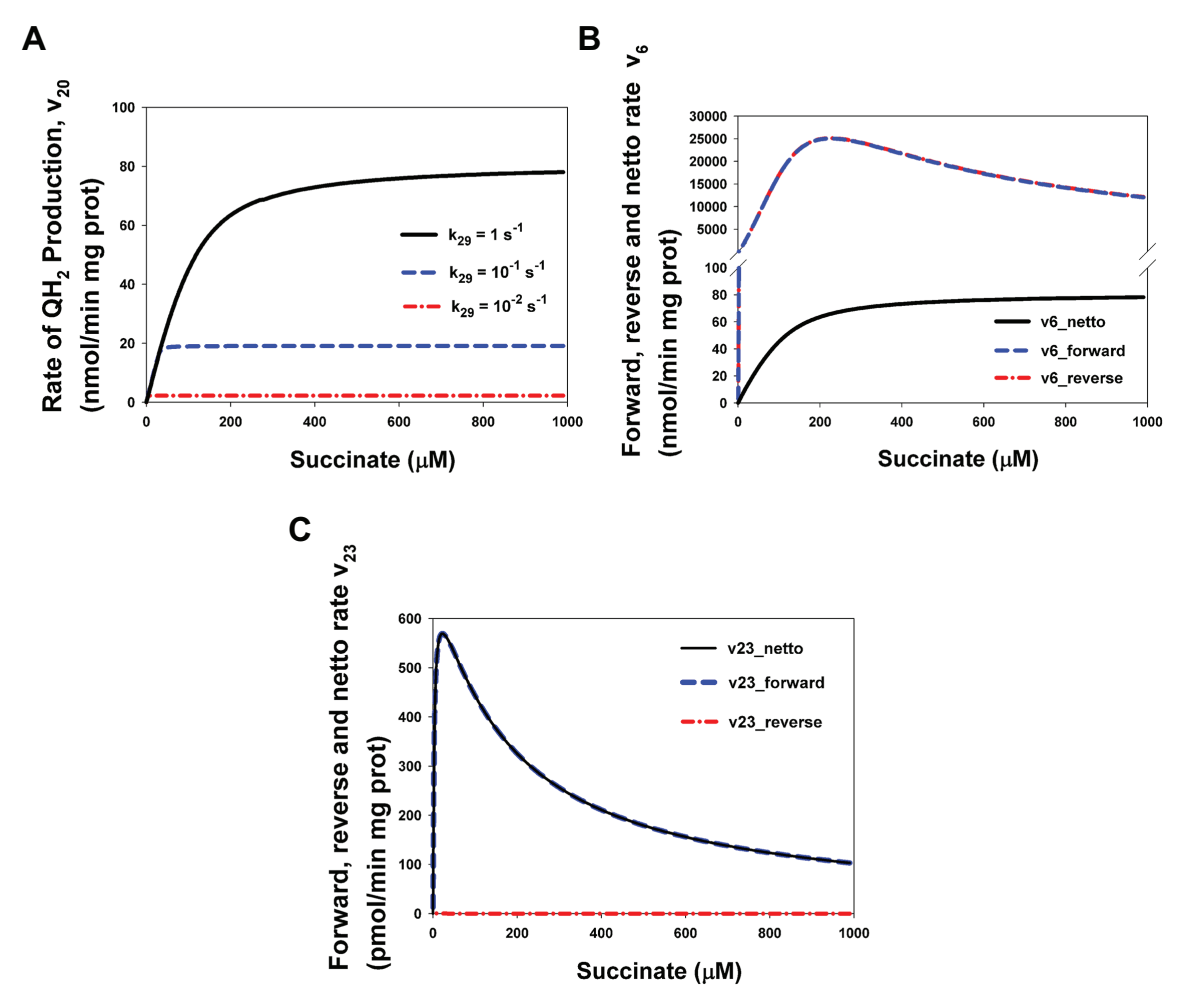
Computer simulated bell-shaped vs. hyperbolic kinetics of ROS production and succinate-Q reductase (SQR) activity of CII. **(A)** The hyperbolic stationary dependence of the QH_2_ production rate, v_20_, on the succinate concentration in the basal state (k_29_ = 1 s^−1^) and upon intermediate (k_29_ = 0.1 s^−1^) and strong (k_29_ = 0.01 s^−1^) inhibition of respiratory Complex III (CIII) that was simulated by decreasing the catalytic constant k_29_ of QH_2_ oxidation. **(B)** Computer simulated dependence of stationary rates of unidirectional mainstream electron flows in forward (from FADH_2_ to oxidized [2Fe-2S] cluster), v_6_forward_, and reverse (from reduced [2Fe-2S]^−^ cluster to FADH^•^), v_6_reverse_, direction and netto rate v_6_netto_ = v_6_forward −_ v_6_reverse_ on the succinate concentration. **(C)** Computer simulated dependence of stationary rates of unidirectional bypass electron flows in forward (from FADH_2_ to O_2_), v_23_forward_, and reverse (from O_2_^−^ to FADH^•^), v_23_reverse_, direction and netto rate v_23_netto_ = v_23_forward −_ v_23_reverse_ on the succinate concentration. Values of the rate constant k_29_ shown in Figure 3A. Black solid curve corresponds to the k_29_ = 1 s^−1^, blue dashed curves – k_29_ = 0.1 s^−1^, and red dash-dot curves – k_29_ = 0.01 s^−1^. The rest model parameter values are presented in Supplementary Table S2. Curves in Figures 3B,C are designated as follows: black solid curves correspond to the netto rates v_6_netto_ **(B)** and v_23_netto_ **(C)**, blue dashed curves – unidirectional forward rates v_6_forward_ **(B)** and v_23_forward_ **(C)**, and red dash-dot curves – unidirectional reverse rates v_6_ reverse_ **(B)** and v_23_ reverse_ **(C)**. All computer simulations were made at model parameter values presented in Supplementary Table S2.

Computer simulation results presented in Figure 5 explain this situation. First, the computer simulated stationary rate v_20_ really has the hyperbolic dependence on the succinate concentration (Figure 5A) and decreases at decreasing k_29_, i.e., decreasing QH_2_ oxidation that results in a suppression of SQR activity. Second, Figures 5B,C show why the rates v_6_ (that equal v_20_ in the steady state) and v_23_ (Figure 1A; Supplementary Table S1) that have one the same substrate and one the same product have qualitatively different kinetics, hyperbolic (v_6_), and bell-shaped (v_23_). Figure 5B shows that unidirectional mainstream electron flows in forward (from FADH_2_ to oxidized [2Fe-2S] cluster), v_6_forward_, and reverse (from reduced [2Fe-2S]^−^ cluster to FADH^•^), v_6_reverse_, direction (expression for them in the section Supplementary Data Mathematical model) really follow nonmonotonic FADH_2_ and FADH^•^ concentration and have bell-shaped kinetics. However, the netto rate: v_6_netto_ = v_6_forward −_ v_6_reverse_ has hyperbolic kinetics due to common-mode inphase changes with a close amplitude in v_6_forward_ and v_6_reverse_ (Figure 5B). Values of these rates v_6_forward_ and v_6_reverse_ are so high compare to v_6_netto_ and close each other that curve for them are indistinguishable in Figure 3B. On the contrary, v_23_reverse_ is very small compare to v_23_forward_ (Figure 5C) due to a small concentration of the one product of this reaction, superoxide O_2_^−^. That is why the netto rate of superoxide O_2_^−^ production by FADH_2_, v_23_netto_ = v_23_forward −_ v_23_reverse_ follows the forward rate v_23_forward_ only and has bell-shaped kinetics.

#### The Effect of Limitation of Succinate Transport on ROS Production by CII

It is worth noting that the succinate concentration required for optimal (peak) ROS generation by CII upon inhibition of CIII or Q-binding site is different in different experiments and changes from approximately 50–500 μM in the experiments with both SMP and intact mitochondria (Quinlan et al., 2012; Siebels and Dröse, 2013; Grivennikova et al., 2017). It was proposed earlier (Grivennikova et al., 2017) that a high optimal succinate concentration up to 400–500 μM in intact mitochondria may be related to limitations of succinate permeability into the mitochondrial matrix, where succinate dehydrogenase active site is located. These limitations were simulated in the model by changes in the rate constant of succinate binding to FAD, k_1_, because diffusion is one of the steps of succinate binding. Computational modeling results on changes in the rate constant k_1_ presented in Supplementary Figure S3 support, in part, this hypothesis. A decrease in k_1_ results in a shift of the optimal succinate concentration to the high values of succinate with simultaneous decrease in the maximal rate of ROS production. These changes resemble the effect of inhibition of CIII and Q-binding site. Therefore, it seems most likely that different succinate optimal values of ROS production observed in different experiments are related to both different power of inhibition of CIII or Q-binding site in different experiments and transport limitations of succinate.

#### The Role of Heme *b* in the Electron Transfer in CII

It should point out that our preliminary computational modeling analysis of the simplified model of assembled CII without heme *b* as an electron carrier in the electron transfer pathway from succinate to Q at the Q-binding site (Markevich et al., 2019) predicts similar effects of inhibition of CIII on ROS production by CII, that is the high-amplitude bell-shaped dependence of the rate of ROS production by CII on the succinate concentration.

**Figure 6:**
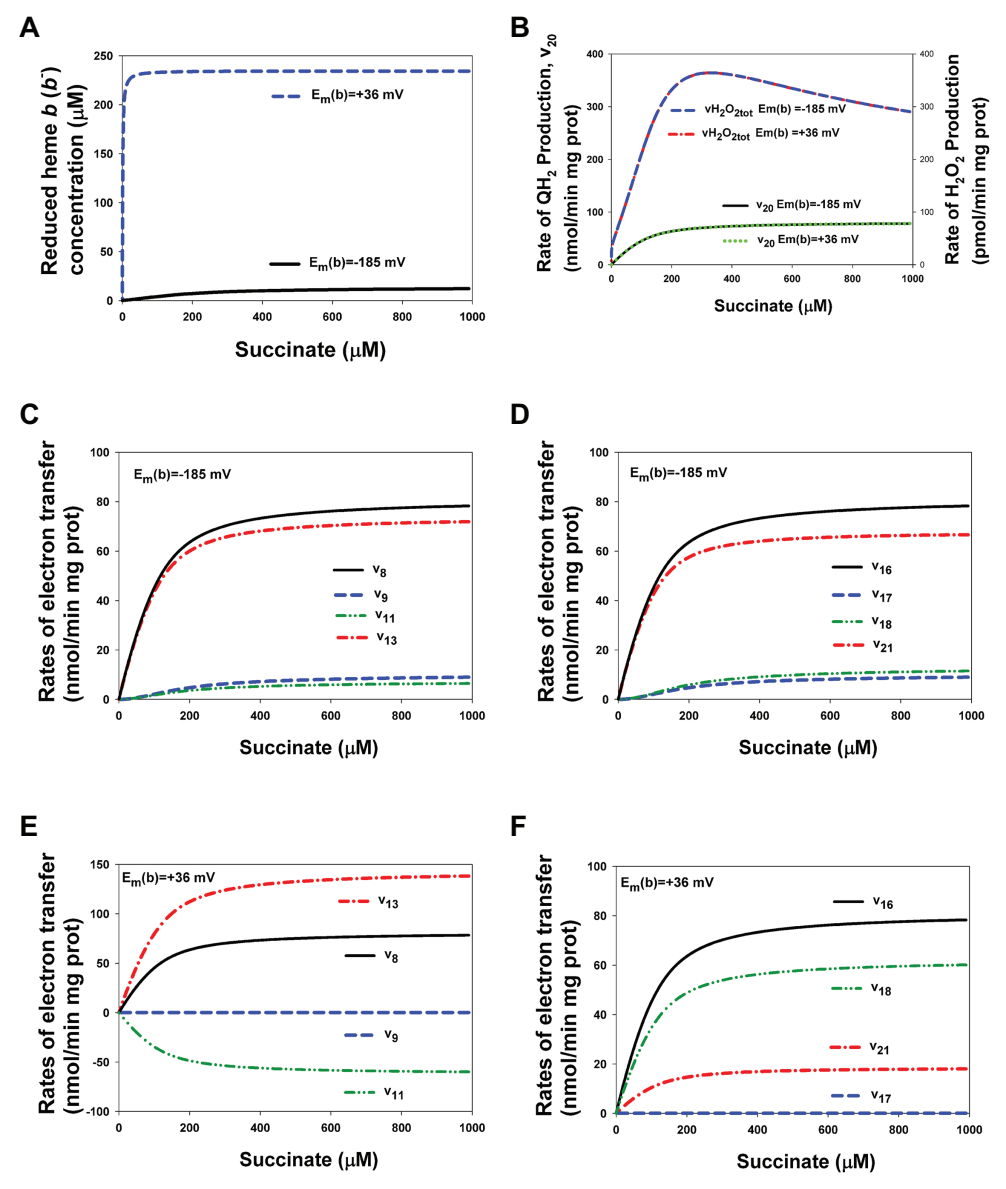
Computer simulated alterations in the reduced heme *b* concentration and rates of electron flows in CII related to changes in the value of heme *b* midpoint potential, E_m_(*b*), from −185 up to +36 mV. **(A)** The dependence of the reduced heme *b*, *b*^−^, concentration on the succinate concentration at two different values of E_m_(*b*), −185 mV (black solid curve) and +36 mV (blue dashed curve). **(B)** The dependence of the rate of QH_2_ production, v_20_, and the total rate of H_2_O_2_ generation by CII, vH_2_O_2tot_, at two different values of E_m_(*b*), −185 mV (black solid and blue dashed curves, respectively) and + 36 mV (green dot and red dash-dot curves, respectively). **(C–F)** Computer simulated dependence of the rates of electron flows from [4Fe-4S] cluster to ubiquinone at ubiquinone (Q)-binding site on the succinate concentration is shown at two different values of E_m_(*b*), −185 mV **(C,D)** and +36 mV **(E,F)** and separately for the first **(C,E)** and second **(D,F)** electron transfer. All computer simulations were made at model parameter values presented in Supplementary Table S2.

Computational analysis of the present extended model with heme *b*, i.e., included the thermodynamic cycle [3Fe-4S]↔heme *b*↔Q↔[3Fe-4S] [reactions (9, 11, and 13) for the first electron and reactions (17, 18, and 21) for the second electron transfer] that was experimentally studied in detail in (Anderson et al., 2014) shows that the value of midpoint potential of the heme *b*, E_m_(*b*), does not affect on the rates of QH_2_ and ROS production by CII in the steady state, although affects on the rates of electron transfer in the thermodynamic cycle including heme *b* (Figure 6). The effect of changes in E_m_(*b*) values from −185 mV used in the present model for bovine heart CII (Supplementary Table S2) up to +36 mV for *Escherichia coli* (Anderson et al., 2014) on the stationary rates of electron transfer in CII and reduction of heme *b* was studied computationally.

Figure 6A shows that the stationary concentration of reduced heme *b*, *b*^−^, increases slowly up to the small concentration, 12 μM, that is about 5% of the total heme *b* concentration that equal to 235 μM, with an increase in the succinate concentration at E_m_(*b*) = −185 mV while increasing E_m_(*b*) up to +36 mV results in almost complete reduction of heme *b,* an increase in *b*^−^ concentration up to 230 μM, or 98% of the total heme *b* concentration, at 40 μM succinate concentration. However, despite a strong difference in the reduced steady-state heme *b* concentration at different E_m_(*b*) values the rates of QH_2_ production, v_20_, i.e., the SQR activity, ROS production, and vH_2_O_2tot_, remain unchanged (Figure 6B).

As to the rates of electron transfer related to the thermodynamic cycle [3Fe-4S]↔heme *b*↔Q↔[3Fe-4S], that is reactions (9, 11, and 13) for the first electron and reactions (17, 18, and 21) for the second electron transfer, the computer simulation results presented in Figures 6C,D for E_m_(*b*) = −185 mV and Figures 6E,F for E_m_(*b*) = +36 mV show that these rates change very much when changing E_m_(*b*). First of all, it should be pointed that input electron flow entering the thermodynamic cycle from [4Fe-4S] cluster, v_8_ for the first electron and v_16_ for the second electron transfer, remain unchanged at different E_m_(*b*) values and equal to the output flow, i.e., the rate of QH_2_ production, v_20_. Other curves in Figures 6C–F describe the rates of electron flows inside the thermodynamic cycle. The most interesting of them is v_11_ at E_m_(*b*) = +36 mV (Figure 6E) that describes the first electron transfer between heme *b* and Q. Computer simulation results predict that the first electron transfer occurs in the reverse direction from semiquinone to heme *b* at the high value of E_m_(*b*) equal to +36 mV. However, this effect is compensated by increasing the first electron flow in forward direction from [3Fe-4S] cluster to Q, v_13_.

It is necessary to emphasize that it was taken into account “the principle of detail balancing” (Hearon, 1953) for thermodynamic cycles at all computations that requires the product of equilibrium constants along a cycle to be equal to 1. For the thermodynamic cycle [3Fe-4S]↔heme *b*↔Q↔[3Fe-4S] this means the following relations: K_eq13_ = K_eq9_·K_eq11_ and K_eq21_ = K_eq17_·K_eq18_. The values of K_eq9_, K_eq11_, and K_eq17_, K_eq18_ at E_m_(*b*) = −185 mV are presented in Supplementary Table S2. Increasing E_m_(*b*) from −185 up to +36 mV results in the following changes in these equilibrium constants: K_eq9_ = K_eq17_ = 5.55∙10^−5^ exp. [(185 + 36) F/RT] = 5.55∙10^−5^∙6,905 = 0.38; K_eq11_ = 2.72/6905 = 3.94∙10^−4^; and K_eq18_ = 3.269∙10^6^/6905 = 473.4.

That is, the values of equilibrium constants K_eq13_ and K_eq21_ kept unchanged while K_eq9_, K_eq11_, and K_eq17_, K_eq18_ are varied with increasing E_m_(*b*) value.

These results imply that a high level of ROS production by CII induced by mutations in SDH cyt *b* (Ishii et al., 1998) is not related with changes in E_m_(*b*). It is most likely as was pointed earlier (Tran et al., 2007; Anderson et al., 2014) that heme *b* plays more the structural role stabilizing CII as a heterotetramer than for catalysis in CII as an electron carrier. That is, the heme *b* plays an important role in assembly CII because as was shown in (Yu et al., 1987), *in vivo*, SDH is anchored to the inner membrane with the cytochrome *b*_560_. Thus, mutations in the cytochrome b large subunit (SDHC), of CII, that induce oxidative stress and lead to apoptosis (see for review Ishii et al., 2007) results in a suppression of the SQR activity of assembled CII due to its disintegration and it is very likely that in this case the dependence of stationary rates of ROS production on the succinate concentration has the same features as upon inhibition of CIII or Q-binding site considered above.

### Assembled and Disintegrated CII

**Figure 7:**
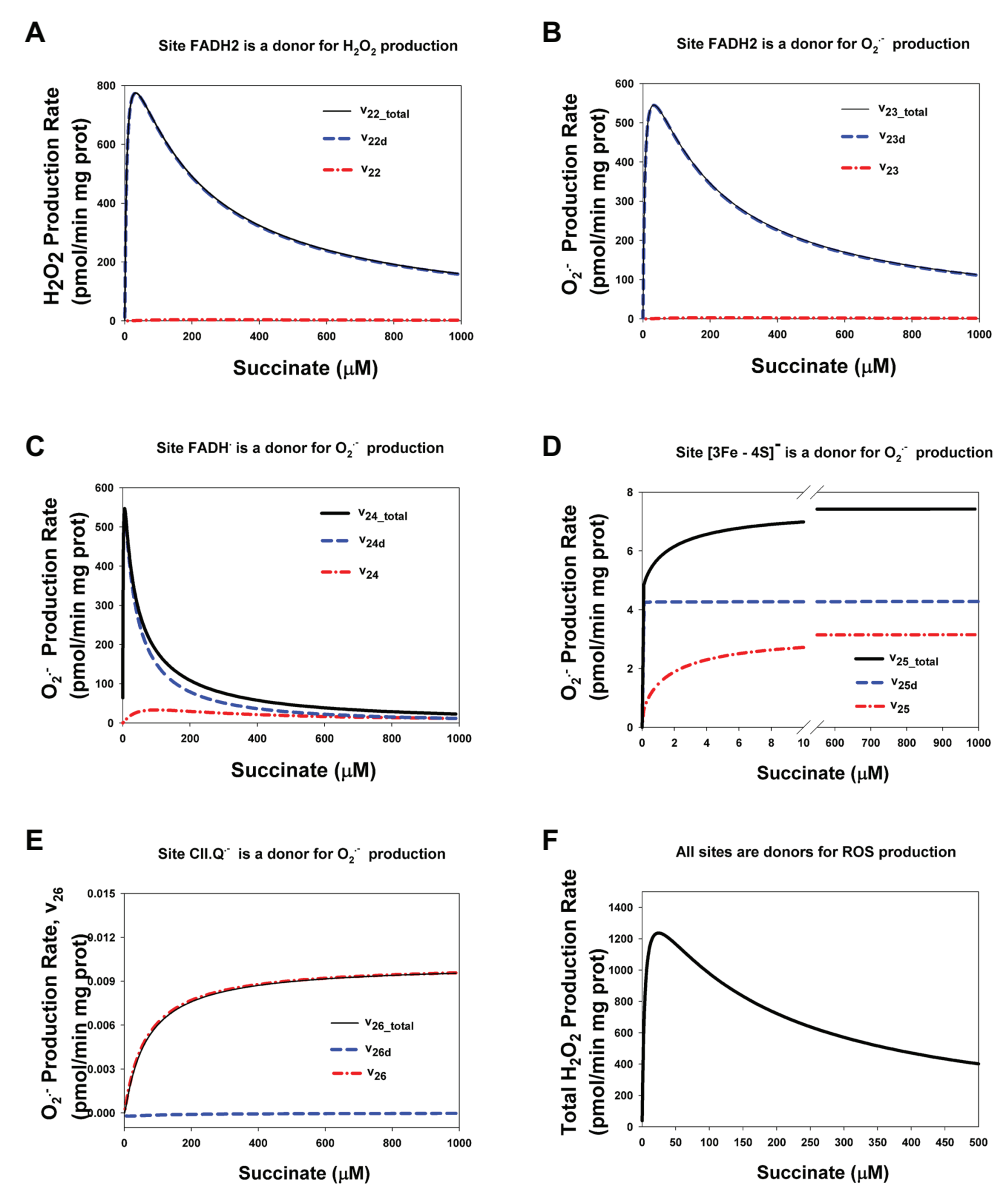
Computer simulated stationary rates of O_2_^−^ and H_2_O_2_ production by different sites in assembled and disintegrated CII. **(A)** The rate of H_2_O_2_ production by FADH_2_ in assembled CII (v_22_), disintegrated CDHA/CDHB subcomplex of CII (v_22d_), and the total rate by both these sites (v_22_total_); **(B–E)** The rates of O_2_^−^ production by the following sites: **(B)** FADH_2_ (v_23_), **(C)** FADH^∙^ (v_24_), **(D)** Reduced [3Fe-4S]^−^ cluster (v_25_), and **(E)** Semiquinone anion binding to CII, site CII.Q^−^ (v_26_). **(F)** The total rate of ROS production by the all redox centers of assembled and disintegrated CII at different values of the concentration of AA5. The total rate of H_2_O_2_ production by CII (vH_2_O_2tot_) that was computed as the rate of H_2_O_2_ release from the mitochondrial matrix to cytosol that equal to the summary rate of H_2_O_2_ production by FADH_2_ in assembled, v_22_, and disintegrated, v_22d_, states and dismutation of O_2_^−^, v_28_, in the matrix at the steady state (see *Explicit functions* in Mathematical model in Supplementary Data). All computer simulations were made at model parameter values as for Figure 2 except AA5 = 0 and the total concentration of CII in the assembled state equal to100 μM and in the disintegrated state ABt = CDt = 135 μM, so the total CII concentration is equal to 235 μM. **(A–E)** Black solid curves correspond to the total ROS production rate for each site in assembled and disintegrated CII, blue dashed curves correspond to the ROS production rate by each site in disintegrated CII, and red dash-dot curves – assembled CII.

CII disintegration resulting in dissociation of the SDHA/SDHB subunits from the membrane-bound SDHC/SDHD complex to the mitochondrial matrix (reviewed in Hwang et al., 2014) and a physical break in electron transfer from succinate to Q at the Q-binding site of CII, i.e., impairment of the SQR activity, causes crucial changes in the kinetics of ROS production by redox centers of these matrix subunits. Comparative analysis of the dependence of the stationary rates of ROS production by different redox centers of CII in assembled and disintegrated states is presented in Figure 7. Here, it was considered the case when a part of CII is in the assembled (CIIt = 100 μM) and a part is in the disintegrated state (ABt = CDt = 135 μM). In addition, it should point those stationary rates of ROS production in assembled CII were computed at simulation the basal state of CII without any inhibitors (all parameter values are presented in Supplementary Table S2). One can see from Figure 7 that the kinetics of ROS production by CII changes from sigmoid in assembled state to the high-amplitude bell-shaped kinetics in disintegrated state. These changes qualitatively and quantitatively close to changes in the kinetics of ROS production by assembled CII upon inhibition of CIII or Q-binding site of CII.

One can see from Figure 7 that the kinetics of ROS production by FADH_2_ (Figures 7A,B) changes from sigmoid in the assembled state (v_22_ and v_23_) to the high-amplitude bell-shaped kinetics in disintegrated state (v_22d_ and v_23d_). These changes qualitatively are the same as the changes in the kinetics of ROS production by FADH_2_ in assembled CII upon inhibition of CIII or Q-binding site of CII (Figures 3A,B; Supplementary Figures S2A,B). Quantitative difference in these changes is related only to different concentration of FADH_2_ in the assembled and disintegrated states. Practically, the same changes from close to sigmoid in the assembled state to the high-amplitude bell-shaped kinetics in the disintegrated state occur in the rate of O_2_^−^ production by FADH^•^ (Figure 7C). So, the total rate of ROS production by FADH_2_ (v_22_total_ = v_22_ + v_22d_) and FADH^•^ (v_23_total_ = v_23_ + v_23d_) in both the assembled and disintegrated states is high amplitude and bell-shaped due to the main contribution of disintegrated FADH_2_ and FADH^•^ to the ROS production rate.

The dependence of the rate of O_2_^−^ production by reduced [3Fe-4S]^−^ cluster on the succinate concentration keeps hyperbolic shape under any condition (Figure 5D) with a decrease in the Michaelis constant in the disintegrated state like in assembled state upon inhibition of the Q-site by AA5 (Figure 3D). And, as expected, the total rate of O_2_^−^ production by semiquinone, CII.Q^−^, at the Q-binding site also keeps hyperbolic shape in the dependence on the succinate concentration (Figure 7E) and includes ROS production in the assembled state only.

The total rate of ROS production by the all sites of assembled and disintegrated CII at different values of the rate constant k_10_ that simulate the effect of AA5 is presented in Figure 7F. The total rate of H_2_O_2_ production by CII (vH_2_O_2tot_) that was computed as the rate of H_2_O_2_ release from the mitochondrial matrix to cytosol is indistinguishable by ROS generation source, assembled, or disintegrated CII, so it is equal to the summary rate of H_2_O_2_ production by FADH_2_ in assembled, v_22_, and disintegrated, v_22d_, states and dismutation of O_2_^−^, v_28_, in the matrix in the steady state (see *Explicit functions* in Mathematical model in the section Supplementary Data). As expected, the maximal rate of ROS production by CII occurs under both conditions, disintegration of CII and upon inhibition of the Q-binding site of assembled CII.

The dependence of stationary rates of ROS production by CII in completely disintegrated state on the succinate concentration is presented in Supplementary Figure S4. Completely disintegrated CII means that all CDHA/CDHB subcomplexes of CII that produce ROS are dissociated from the membrane and located only in the matrix with the concentration of 235 μM. All of dependencies of the ROS production rate by different sites of completely disintegrated CII on the succinate concentration match those in the assembled CII upon a strong inhibition of CIII and the Q-binding site.

Thus, these computer simulation results predict that CII disintegration results in the same changes in the kinetics of ROS production by CII as a suppression of SQR activity of assembled CII upon inhibition of the Q-binding site and/or CIII as was pointed earlier in (Siebels and Dröse, 2013) and induces the high-amplitude bell-shaped dependence of the rate of ROS production by FADH_2_ in the unoccupied dicarboxylate state and FADH^•^ with a shift of the maximal rate to small subsaturated concentration of succinate.

## Conclusion

A computational, mechanistic model of electron transfer and the formation of superoxide (O_2_^−^) and hydrogen peroxide (H_2_O_2_) in CII in the assembled and disintegrated states was developed in the present study to facilitate quantitative analysis of mitochondrial ROS production. The model was calibrated by fitting the computer simulated results to experimental data obtained on SMP prepared from bovine heart mitochondria upon inhibition of Q-binding site by AA5 (Siebels and Dröse, 2013) and from rat heart mitochondria upon inhibition of Complex III by myxothiazol (Grivennikova et al., 2017).

The present computational modeling study predicts that a suppression of the SQR activity of CII resulting from inhibition of CIII or Q-binding site of CII as was pointed earlier in (Siebels and Dröse, 2013) as well as CII disintegration reviewed in (Hwang et al., 2014) causes transition in the succinate-dependence of ROS production from small-amplitude sigmoid (hyperbolic) determined by Q-binding site or/and [3Fe-4S] cluster to the high-amplitude bell-shaped kinetics with a shift to small subsaturated concentration of succinate determined by FADH_2_ in the unoccupied dicarboxylate state and FADH^•^.

Computer simulation results confirm previous hypothesis (Quinlan et al., 2012; Siebels and Dröse, 2013) that the main contribution to the total rate of ROS production by CII upon inhibition of the Q-binding site or/and CIII give unoccupied FADH_2_ and FADH^•^.

The dependence of the rates of ROS production by disintegrated matrix SDHA/SDHB subcomplexes on the succinate concentration matches qualitatively and quantitatively to those in the assembled state upon inhibition of the CII Q-binding site or/and CIII.

It is very likely that semiquinone at the Q-binding site and [3Fe-4S] cluster give a small contribution to ROS production in both basal and inhibited state. Presented modeling results show that the dependence of the rates of ROS production by these redox centers on the succinate concentration keeps hyperbolic shape with very small maximal rate due to the small values of the catalytic constants of ROS formation in any state of CII.

Computational modeling analysis of the model included the thermodynamic cycle [3Fe-4S]↔heme *b*↔Q↔[3Fe-4S] shows that the value of midpoint potential of the heme *b*, E_m_(*b*), does not affect on the rates of QH_2_ and ROS production by CII in the steady state, although affects on the heme *b* reduction and the rates of electron transfer in the thermodynamic cycle including heme *b*. This result confirms theoretically suggestions pointed earlier (Tran et al., 2007; Anderson et al., 2014) that heme *b* plays more the structural role stabilizing CII as a heterotetramer than for catalysis in CII as an electron carrier.

Thus, the results of this work allow us to evaluate the catalytic constants of ROS formation by each of the redox centers of CII and predict their contribution to the overall generation of ROS by CII in various (basal or inhibited as well as assembled or disintegrated) states of CII. We hope that this will help resolve the many years of debate about which of the CII redox centers are actually involved in the ROS formation. These theoretical predictions are particularly valuable now that experimental methods for directly measuring these catalytic constants have not yet been developed.

Theoretical results on ROS production by disintegrated matrix SDHA/SDHB subcomplexes with bell-shaped dependence on succinate concentration are extremely useful for understanding autocatalytic MPT due to efflux of succinate from mitochondria through the open MPT pore found recently in cardiac mitochondria (Korge et al., 2017). In this case, activation of ROS production by CII at low succinate concentration results in activation of MPT with following activation of apoptosis or necrosis/necroptosis.

## Data Availability Statement

The raw data supporting the conclusions of this article will be made available by the authors, without undue reservation.

## Author Contributions

NM and JH conceived and supervised the study. NM and LM performed computations. NM and JH wrote this report. All authors contributed to the article and approved the submitted version.

### Conflict of Interest

The authors declare that the research was conducted in the absence of any commercial or financial relationships that could be construed as a potential conflict of interest.

## Abbreviations

ROS: Reactive oxygen species
CII: Respiratory Complex II
CIII: Respiratory Complex III
FAD: Flavin adenine dinucleotide
SDH: Succinate dehydrogenase
Q: Ubiquinone
SQR: Succinate-Q reductase
SMP: Submitochondrial particles
