# Supplementary material for: Computational Modeling Analysis of Generation of Reactive Oxygen Species by Mitochondrial Assembled and Disintegrated Complex II
Source: Front Physiol. 2020 Oct 16;11:557721. doi: 10.3389/fphys.2020.557721 (PMC7596731; doi:10.3389/fphys.2020.557721)
Supplement: Supplementary file 1 [file Table_1.docx]

**Supplementary Data**

**Computational modeling analysis of generation of reactive oxygen species by mitochondrial assembled and disintegrated Complex II.**

Nikolay I. Markevich ^1^*, Lubov N. Markevich ^2^, Jan B. Hoek ^3^

^1^Institute of Theoretical and Experimental Biophysics RAS, Pushchino, Moscow region, 142290, Russian Federation

^2^Institute of Cell Biophysics of RAS, Pushchino, Moscow region, 142290, Russian Federation

^3^MitoCare Center for Mitochondrial Research, Department of Pathology, Anatomy, and Cell

Biology, Thomas Jefferson University, Philadelphia, PA 19107, USA

*Corresponding author.

Present address for correspondence:

Nikolay I. Markevich

Institute of Theoretical and Experimental Biophysics, Russian Academy of Sciences,

3 Institutskaya street,

Pushchino, Moscow region 14290, Russia

Email: [markevich.nick@gmail.com](mailto:markevich.nick@gmail.com)

Phone: +7 9160983229

**Supplementary Tables**

Supplementary Tables S1 and S2 presented below correspond to the kinetic schemes of elecron transfer in Figure 1 in the main text and describe in detail chemical reactions, rate equations and parameter values of all stages of electron transfer and formation of reactive oxygen species in Complex II.

**Supplementary Table S1. Reactions and rate equations in Assembled and Disintegrated Complex II.**

| No | Reaction | Rate equation |
| --- | --- | --- |
| **Reactions and rate equations in Assembled Complex II** | | |
| **Oxidation of succinate to fumarate** | | |
| 1 | FAD + suc = FAD.suc | V_1_=k_1_ ∙ ( FAD ∙ suc – FAD.suc / K_eq1_) |
| 2 | FAD.suc = FADH2.fum | V_2_=k_2_ ∙ ( FAD.suc – FADH2.fum/K_eq2_) |
| 3 | FADH2.fum = FADH2 + fum | V_3_=k_3_ ∙ ( FADH2.fum – FADH2 ∙ fum / K_eq3_) |
| 4 | FAD + fum = FAD.fum | V_4_=k_4_ ∙ ( FAD ∙ fum - FAD.fum / K_eq4_) |
| 5 | FADH2 + suc = FADH2.suc | V_5_=k_5_ ∙ ( FADH2 ∙ suc – FADH2.suc / K_eq5_) |
| **The first electron transfer** | | |
| 6 | FADH2 + [2Fe-2S] = FADH^∙^ + [2Fe-2S]^-^ + H^+^ | V_6_=k_6_ ∙ ( FADH2 ∙ [2Fe-2S] – FADH^∙^ ∙ [2Fe-2S]^-^ ∙ H^+^ / K_eq6_) |
| 7 | [2Fe-2S]^-^ + [4Fe-4S] = [2Fe-2S] + [4Fe-4S]^-^ | V_7_=k_7_ ∙ ([2Fe-2S]^-^ ∙ [4Fe-4S] – [2Fe-2S] ∙ [4Fe-4S]^-^/ K_eq7_) |
| 8 | [4Fe-4S]^-^ + [3Fe-4S] = [4Fe-4S] + [3Fe-4S]^-^ | V_8_=k_8_ ∙ ([4Fe-4S]^-^ ∙ [3Fe-4S] – [4Fe-4S] ∙ [3Fe-4S]^-^/ K_eq8_) |
| 9 | [3Fe-4S]^-^ + b = [3Fe-4S] + b^-^ | V_9_=k_9_ ∙ ([3Fe-4S]^-^ ∙ b – [3Fe-4S] ∙ b^-^ / K_eq9_) |
| 10 | CII + Q = CII.Q | V_10_=k_10_ ∙ ( CII ∙ Q – CII.Q / K_eq10_) |
| 11 | CII.Q + b^-^ = CII.Q**^.^**^-^ + b | V_11_=k_11_ ∙ (CII.Q ∙ b^-^– CII.Q**^.^**^-^ ∙ b / K_eq11_) |
| 12 | CII.Q**^.^**^-^ + H^+^ = CII.QH**^.^** | V_12_=k_12_ ∙ (CII.Q**^.^**^-^ ∙ H^+^ – CII.QH**^.^** / K_eq12_) |
| 13 | CII.Q + [3Fe-4S]^-^ = CII.Q**^.^**^-^ + [3Fe-4S] | V_13_=k_13_ ∙ (CII.Q ∙ [3Fe-4S]^-^ – CII.Q**^.^**^-^ ∙ [3Fe-4S] / K_eq13_) |
| **The second electron transfer** | | |
| 14 | FADH^∙^ + [2Fe-2S] = FAD + [2Fe-2S]^-^ + H^+^ | V_14_=k_14_ ∙ (FADH^∙^ ∙ [2Fe-2S] – FAD ∙ [2Fe-2S]^-^ ∙ H^+^ / K_eq14_) |
| 15 | [2Fe-2S]^-^ + [4Fe-4S] = [2Fe-2S] + [4Fe-4S]^-^ | V_15_=k_15_∙ ([2Fe-2S]^-^ ∙ [4Fe-4S] – [2Fe-2S] ∙ [4Fe-4S]^-^/ K_eq15_) |
| 16 | [4Fe-4S]^-^ + [3Fe-4S] = [4Fe-4S] + [3Fe-4S]^-^ | V_16_=k_16_ ∙ ([4Fe-4S]^-^ ∙ [3Fe-4S] – [4Fe-4S] ∙ [3Fe-4S]^-^/ K_eq16_) |
| 17 | [3Fe-4S]^-^ + b = [3Fe-4S] + b^-^ | V_17_=k_17_ ∙ ([3Fe-4S]^-^ ∙ b – [3Fe-4S] ∙ b^-^ / K_eq17_) |
| 18 | CII.QH**^.^** + b^-^ = CII.QH^-^ + b | V_18_=k_18_ ∙ (CII.QH**^.^** ∙ b^-^– CII.QH^-^ ∙ b / K_eq18_) |
| 19 | CII.QH^-^ + H^+^ = CII.QH2 | V_19_=k_19_ ∙ (CII.QH^-^ ∙ H^+^ – CII.QH2) / K_eq19_) |
| 20 | CII.QH2 = CII + QH2 | V_20_=k_20_ ∙ (CII.QH2 – CII ∙ QH2 / K_eq20_) |
| 21 | CII.QH**^.^** + [3Fe-4S]^-^ = CII.QH^-^ + [3Fe-4S] | V_21_=k_21_ ∙ (CII.QH**^.^** ∙ [3Fe-4S]^-^ – CII.QH^-^ ∙ [3Fe-4S] / K_eq21_) |
| **Hydrogen peroxide (H_2_O_2_) production by Assembled Complex II** | | |
| 22 | FADH2 + O_2_ = FAD + H_2_O_2_ | V_22_=k_22_ ∙ (FADH2 ∙ O_2_ – FAD ∙ H_2_O_2_ / K_eq22_) |
| **Superoxide anion (O_2_^.-^) production by Assembled Complex II** | | |
| 23 | FADH2 + O_2_ = FADH^∙^ + O_2_**^.^**^-^ + H^+^ | V_23_=k_23_ ∙ (FADH2 ∙ O_2_ – FADH^∙^ ∙ O_2_**^.^**^-^ ∙ H^+^ / K_eq23_) |
| 24 | FADH^∙^ + O_2_ = FAD + O_2_**^.^**^-^ +H^+^ | V_24_=k_24_ ∙ (FADH^∙^ ∙ O_2_ – FAD ∙ O_2_**^.^**^-^ ∙ H^+^ / K_eq24_) |
| 25 | [3Fe-4S]^-^+ O_2_=[3Fe-4S] + O_2_**^.^**^-^ | V_25_=k_25_ ∙ ([3Fe-4S]^-^ ∙ O_2_ – [3Fe-4S] ∙ O_2_**^.^**^-^ / K_eq25_) |
| 26 | CII.Q**^.^**^-^ + O_2_ = CII.Q + O_2_**^.^**^-^ | V_26_=k_26_ ∙ (CII.Q**^.^**^-^ ∙ O_2_ – CII.Q ∙ O_2_**^.^**^-^ / K_eq26_) |
| **Competitive inhibition of the Q-site by atpenin A5 (AA5)** | | |
| 27 | CII + AA5 = CII.AA5 | V_27_=k_27_ ∙ ( CII ∙ AA5 – CII.AA5 / K_eq27_) |
| **Accompanying reactions in the matrix and inner membrane** | | |
| **Superoxide anion dismutation in the mitochondrial matrix** | | |
| 28 | 2 O_2_**^.^**^-^ + 2H^+^ → O_2_ + H_2_O_2_ | V_28_=V_max28_ ∙ O_2_**^.^**^-^ / (K_m28_ + O_2_**^.^**^-^) |
| **Ubiquinol (QH2) oxidation in the mitochondrial inner membrane** | | |
| 29 | QH2 → Q + 2H^+^ | V_29_= k_29_ ∙ QH2 |
| **Release of hydrogen peroxide (H_2_O_2_) from the mitochondrial matrix to cytosol** | | |
| 30 | H_2_O_2_ → | V_30_= k_30_ ∙ H_2_O_2_ |
| **Complex II assembly/disintegration** | | |
| 31 | ABt + CDt = CIIt | V_31_=k_31_ ∙ (ABt ∙ CDt – CIIt / Keq_31_) |
| **Reactions and rate equations in Disintegrated SDHA/SDHB subunits of Complex II** | | |
| **Oxidation of succinate to fumarate** | | |
| 1d | FADd + suc = FADd.suc | V_1d_=k_1d_ ∙ ( FADd ∙ suc – FADd.suc / K_eq1d_) |
| 2d | FADd.suc = FADH2d.fum | V_2d_=k_2d_ ∙ ( FADd.suc – FADH2d.fum / K_eq2d_) |
| 3d | FADH2d.fum=FADH2d + fum | V_3d_=k_3d_ ∙ ( FADH2d.fum – FADH2d ∙ fum / K_eq3d_) |
| 4d | FADd + fum = FADd.fum | V_4d_=k_4d_ ∙ ( FADd ∙ fum — FADd.fum / K_eq4d_) |
| 5d | FADH2d + suc = FADH2d.suc | V_5d_=k_5d_ ∙ ( FADH2d ∙ suc – FADH2d.suc / K_eq5d_) |
| **The first electron transfer** | | |
| 6d | FADH2d + [2Fe-2S]d = FADHd^∙^ + [2Fe-2S]d^-^ + H^+^ | V_6d_=k_6d_ ∙ ( FADH2d ∙ [2Fe-2S]d – FADHd^∙^ ∙ [2Fe-2S]d^-^∙ H^+^ / K_eq6d_) |
| 7d | [2Fe-2S]d^-^ + [4Fe-4S]d = [2Fe-2S]d + [4Fe-4S]d^-^ | V_7d_=k_7d_ ∙ ([2Fe-2S]d^-^ ∙ [4Fe-4S]d – [2Fe-2S]d ∙ [4Fe-4S]d^-^ / K_eq7d_) |
| 8d | [4Fe-4S]d^-^ + [3Fe-4S]d = [4Fe-4S]d + [3Fe-4S]d^-^ | V_8d_=k_8d_ ∙ ([4Fe-4S]d^-^ ∙ [3Fe-4S]d – [4Fe-4S]d ∙ [3Fe-4S]d^-^ / K_eq8d_) |
| **The second electron transfer** | | |
| 14d | FADHd^∙^ + [2Fe-2S]d = FADd + [2Fe-2S]d^-^ + H^+^ | V_14d_=k_14d_ ∙ (FADHd^∙^ ∙ [2Fe-2S]d – FADd ∙ [2Fe-2S]d^-^ ∙ H^+^ / K_eq14d_) |
| 15d | [2Fe-2S]d^-^ + [4Fe-4S]d = [2Fe-2S]d + [4Fe-4S]d^-^ | V_15d_=k_15d_∙ ([2Fe-2S]d^-^ ∙ [4Fe-4S]d – [2Fe-2S]d ∙ [4Fe-4S]d^-^ / K_eq15d_) |
| 16d | [4Fe-4S]d^-^ + [3Fe-4S]d = [4Fe-4S]d + [3Fe-4S]d^-^ | V_16d_=k_16d_ ∙ ([4Fe-4S]d^-^ ∙ [3Fe-4S]d – [4Fe-4S]d ∙ [3Fe-4S]d^-^/ K_eq16d_) |
| **Hydrogen peroxide (H_2_O_2_) production by Disintegrated SDHA/SDHB subunits** | | |
| 22d | FADH2d + O_2_ = FADd + H_2_O_2_ | V_22d_=k_22d_ ∙ (FADH2d ∙ O_2_ – FADd ∙ H_2_O_2_ / K_eq22d_) |
| **Superoxide anion (O_2_^.-^) production by Disintegrated SDHA/SDHB subunits** | | |
| 23d | FADH2d + O_2_ = FADHd^∙^ + O_2_**^.^**^-^ + H^+^ | V_23d_=k_23d_ ∙ (FADH2d ∙ O_2_ – FADHd^∙^ ∙ O_2_**^.^**^-^ ∙ H^+^ / K_eq23d_) |
| 24d | FADHd^∙^ + O_2_ = FADd + O_2_**^.^**^-^ + H^+^ | V_24d_=k_24d_ ∙ (FADHd^∙^ ∙ O_2_ – FADd ∙ O_2_**^.^**^-^ ∙ H^+^ / K_eq24d_) |
| 25d | [3Fe-4S]d^-^ + O_2_ = [3Fe-4S]d + O_2_**^.^**^-^ | V_25d_=k_25d_ ∙ ([3Fe-4S]d^-^ ∙ O_2_ – [3Fe-4S]d ∙ O_2_**^.^**^-^ / K_eq25d_) |
| **Reactions and rate equations in Disintegrated SDHC/SDHD subunits of Complex II** | | |
| **Electron transfer in Disintegrated SDHC/SDHD subunits** | | |
| 10d | CD + Q = CD.Q | V_10d_=k_10d_ ∙ ( CD ∙ Q – CD.Q / K_eq10d_) |
| 11d | CD.Q + b^-^ = CD.Q**^.^**^-^ + b | V_11d_=k_11d_ ∙ (CD.Q ∙ b^—^CD.Q**^.^**^-^ ∙ b / K_eq11d_) |
| 12d | CD.Q**^.^**^-^ + H^+^ = CD.QH**^.^** | V_12d_=k_12d_ ∙ (CD.Q**^.^**^-^ ∙ H^+^ – CD.QH**^.^** / K_eq12d_) |
| 18d | CD.QH**^.^** + b^-^ =CD.QH^-^ + b | V_18d_=k_18d_ ∙ (CD.QH**^.^** ∙ b^—^CD.QH^-^ ∙ b / K_eq18d_) |
| 19d | CD.QH^-^ + H^+^ = CD.QH2 | V_19d_=k_19d_ ∙ (CD.QH^-^ ∙ H^+^ – CD.QH2 / K_eq19d_) |
| 20d | CD.QH2 = CD + QH2 | V_20d_=k_20d_ ∙ (CD.QH2 – CD ∙ QH2 / K_eq20d_) |
| **Superoxide anion (O_2_^.-^) production by Disintegrated SDHC/SDHD subunits** | | |
| 26d | CD.Q**^.^**^-^ + O_2_ = CD.Q + O_2_**^.^**^-^ | V_26d_=k_26d_ ∙ (CD.Q**^.^**^-^ ∙ O_2_ – CD.Q ∙ O_2_**^.^**^-^ / K_eq26d_) |

*Note.* CIIt, ABt and CDt are the total concentrations of assembled complex II (CII), SDHA/SDHB and SDHC/SDHD subunits in disintegrated Complex II, respectively.

Supplementary Table S2. Parameter values for the model.

| Reac-tion  No | Midpoint potential  E_m_=E, (mV) | Equilibrium constant  K_eq_ | k_forward_ | Other parameters | | Reference |
| --- | --- | --- | --- | --- | --- | --- |
| **Reactions and rate equations in Assembled Complex II** | | | | | | |
| **Oxidation of succinate to fumarate** | | | | | | |
| 1 |  | 0.1 µM^-1^ | 0.05 µM^-1^⋅s^-1^ |  | | [1]^b^ |
| 2 |  | 3.6∙10^-4d^ | 1∙10^3^ s^-1^ |  | |  |
| 3 |  | 50 µM | 5∙10^4^ s^-1^ |  | | [1]^b^ |
| 4 |  | 0.004 µM^-1^ | 1 µM^-1^⋅s^-1^ |  | | [1]^b^ |
| 5 |  | 6.25∙10^-3^ µM^-1^ | 1 µM^-1^⋅s^-1^ |  | | [1]^b^ |
| **The first electron transfer** | | | | | | |
| 6 | E(FADH**^∙^/** FADH2) = -31  E([2Fe-2S]) = 0 | 3.456 µM | 10^4^ µM^-1^⋅s^-1^ | | pH = 7  pH = 7.4 | [2]^a^  [3]^a^ |
| 7 | E([2Fe-2S]) = 0  E([4Fe-4S]) = -260 | 3.04∙10^-5^ | 10^4^ µM^-1^⋅s^-1^ | | pH = 7.4 | [3]^a^ |
| 8 | E([4Fe-4S]) = -260  E([3Fe-4S]) = 60 | 3.6∙10^5^ | 10^4^ µM^-1^⋅s^-1^ | | pH = 7.4 | [3]^a^  [4]^a^ |
| 9 | E([3Fe-4S]) = 60  E(b)=-185 | 5.55∙10^-5^ | 843 µM^-1^⋅s^-1^ | |  | [4,5]^a^  [6]^c^ |
| 10 |  | 3.33 µM^-1^ | 10 µM^-1^⋅s^-1^ | |  | [7]^b^ |
| 11 | E(b)=-185  E(Q/Q^.-^) = -160 | 2.72 | 2.4⋅10^4^ µM^-1^⋅s^-1^ | | pH = 7 | [5,8]^a^  [6]^c^ |
| 12 |  | 0.204 µM^-1e^ | 10^3^ µM^-1^⋅s^-1^ | | pH = 7 | [9]^b^ |
| 13 | E([3Fe-4S]) = 60  E(Q/Q^.-^) = -160 | 1.5∙10^-4^ | 7.2⋅10^3^ µM^-1^⋅s^-1^ | | pH = 7.4  pH = 7 | [4,8]^a^  [6]^c^ |
| **The second electron transfer** | | | | | | |
| 14 | E(FAD**/** FADH**^∙^**) = -127  E([2Fe-2S]) = 0 | 166.77 µM | 10^4^ µM^-1^⋅s^-1^ | | pH = 7  pH = 7.4 | [2,3]^a^ |
| 15 | E([2Fe-2S]) = 0  E([4Fe-4S]) = -260 | 3.04∙10^-5^ | 10^4^ µM^-1^⋅s^-1^ | | pH = 7.4 | [3]^a^ |
| 16 | E([4Fe-4S]) = -260  E([3Fe-4S]) = 60 | 3.6∙10^5^ | 10^4^ µM^-1^⋅s^-1^ | | pH = 7.4 | [3]^a^  [4]^a^ |
| 17 | E([3Fe-4S]) = 60  E(b)=-185 | 5.55∙10^-5^ | 843 µM^-1^⋅s^-1^ | | pH = 7.4  pH = 7 | [4,5]^a^  [6]^c^ |
| 18 | E(b)=-185  E(QH^.^/QH^-^) = 190 | 3.269∙10^6^ | 2.4⋅10^4^ µM^-1^⋅s^-1^ | |  | [5,8]^a^  [6]^c^ |
| 19 |  | 7.24∙10^4^ µM^-1e^ | 10^3^ µM^-1^⋅s^-1^ | | pH = 7 | [9]^b^ |
| 20 |  | 0.9 µM | 10^3^ s^-1^ | |  | [7]^b^ |
| 21 | E([3Fe-4S]) = 60  E(QH^.^/QH^-^) = 190 | 181.27 | 7.2⋅10^3^ µM^-1^⋅s^-1^ | | pH = 7.4 | [4,8]^a^  [6]^c^ |
| **Hydrogen peroxide (H_2_O_2_) production by Assembled Complex II** | | | | | | |
| 22 | E(O_2_/Н_2_O_2_)= 690  E(FAD**/** FADH2) = -79 | 5.2∙10^26^ | 0.01 µM^-1^⋅s^-1^ | | pH = 7 | [2]^a^ |
| **Superoxide anion (O_2_^.-^) production by Assembled Complex II** | | | | | | |
| 23 | E(O_2_/O_2_^.-^) = -160  E(FADH**^∙^/** FADH2) = -31 | 6∙10^-3^ µM | 0.01 µM^-1^⋅s^-1^ | | pH = 7 | [10]^a^  [2]^a^ |
| 24 | E(O_2_/O_2_^.-^) = -160  E(FAD**/** FADH**^∙^**) = -127 | 0.267 | 0.1 µM^-1^⋅s^-1^ | | pH = 7  pH = 7.4 | [10]^a^  [2]^a^ |
| 25 | E(O_2_/O_2_^.-^) = -160  E([3Fe-4S]) = 60 | 1.5∙10^-4^ | 1⋅10^-3^ µM^-1^⋅s^-1^ | | pH = 7  pH = 7.4 | [10]^a^  [4]^a^ |
| 26 | E(O_2_/O_2_^.-^) = -160  E(Q/Q^.-^) = -160 | 1 | 0.05 µM^-1^⋅s^-1^ | |  | [10]^a^  [8]^a^ |
| **Competitive inhibition of the Q-site by atpenin A5 (AA5)** | | | | | | |
| 27 |  | 2⋅10^5^ µM^-1h^ | 10 µM^-1^⋅s^-1h^ | |  | Adjusted values |
| **Accompanying reactions in the matrix and inner membrane** | | | | | | |
| **Superoxide anion dismutation in the mitochondrial matrix** | | | | | | |
| 28 |  |  |  | V_max28_ = 5.6 ∙ 10^4^ µM⋅s^-1f^  K_m28_ = 50 µM | | [11]^d^ |
| **Ubiquinol (QH2) oxidation in the mitochondrial inner membrane** | | | | | | |
| 29 |  |  | 1 s^-1^ |  | |  |
| **Efflux of hydrogen peroxide (H_2_O_2_) from the mitochondrial matrix to cytoplasm** | | | | | | |
| 30 |  |  | 30 s^-1^ |  | | [12]^c^ |
| **Complex II assembly/disintegration** | | | | | | |
| 31 |  | 1 µM^-1^ | 1 µM^-1^⋅s^-1^ |  | | Arbitrary values^g^ |
| **Reactions and rate equations in Disintegrated SDHA/SDHB subunits of Complex II** | | | | | | |
| **Oxidation of succinate to fumarate** | | | | | | |
| 1d |  | 0.1 µM^-1^ | 0.05 µM^-1^⋅s^-1^ |  | | [1]^b^ |
| 2d |  | 3.6∙10^-4г^ | 1∙10^3^ s^-1^ |  | |  |
| 3d |  | 50 µM | 5∙10^4^ s^-1^ |  | | [1]^b^ |
| 4d |  | 0.004 µM^-1^ | 1 µM^-1^⋅s^-1^ |  | | [1]^b^ |
| 5d |  | 6.25∙10^-3^ µM^-1^ | 1 µM^-1^⋅s^-1^ |  | | [1]^b^ |
| **The first electron transfer** | | | | | | |
| 6d | E(FADH**^∙^/** FADH2) =  -31  E([2Fe-2S]) = 0 | 3.456 | 10^4^ µM^-1^⋅s^-1^ | pH = 7  pH = 7.4 | | [2,3]^a^ |
| 7d | E([2Fe-2S]) = 0  E([4Fe-4S]) = -260 | 3.04∙10^-5^ | 10^4^ µM^-1^⋅s^-1^ | pH = 7.4 | | [3]^a^ |
| 8d | E([4Fe-4S]) = -260  E([3Fe-4S]) = 60 | 3.6∙10^5^ | 10^4^ µM^-1^⋅s^-1^ | pH = 7.4 | | [3]^a^  [4]^a^ |
| **The second electron transfer** | | | | | | |
| 14d | E(FAD**/** FADH**^∙^**) =  -127  E([2Fe-2S]) = 0 | 166.77 | 10^4^ µM^-1^⋅s^-1^ | pH = 7  pH = 7.4 | | [2]^a^  [3]^a^ |
| 15d | E([2Fe-2S]) = 0  E([4Fe-4S]) = -260 | 3.04∙10^-5^ | 10^4^ µM^-1^⋅s^-1^ | pH = 7.4 | | [3]^a^ |
| 16d | E([4Fe-4S]) = -260  E([3Fe-4S]) = 60 | 3.6∙10^5^ | 10^4^ µM^-1^⋅s^-1^ | pH = 7.4 | | [3]^a^  [4]^a^ |
| **Hydrogen peroxide (H_2_O_2_) production by Disintegrated SDHA/SDHB subunits** | | | | | | |
| 22d | E(O_2_/Н_2_O_2_)= 690  E(FAD**/** FADH2) = - 79 | 5.2∙10^26^ | 0.01 µM^-1^⋅s^-1^ | pH = 7 | | [2]^a^ |
| **Superoxide anion (O_2_^.-^) production by Disintegrated SDHA/SDHB subunits** | | | | | | |
| 23d | E(O_2_/O_2_^.-^) = -160  E(FADH**^∙^/** FADH2) = - 31 | 6∙10^-3^ | 0.01 µM^-1^⋅s^-1^ | pH = 7 | | [10]^a^  [2]^a^ |
| 24d | E(O_2_/O_2_^.-^)=  -160  E(FAD**/** FADH**^∙^**) =  -127 | 0.267 | 0.1 µM^-1^⋅s^-1^ | pH = 7  pH = 7.4 | | [10]^a^  [2]^a^ |
| 25d | E(O_2_/O_2_^.-^) = -160  E([3Fe-4S]) = 60 | 1.5∙10^-4^ | 1⋅10^-3^ µM^-1^⋅s^-1^ | pH = 7  pH = 7.4 | | [10]^a^    [4]^a^ |
| **Reactions and rate equations in Disintegrated SDHC/SDHD subunits of Complex II** | | | | | | |
| **Electron transfer in Disintegrated SDHC/SDHD subunits** | | | | | | |
| 10d |  | 3.33 µM^-1^ | 10 µM^-1^⋅s^-1^ |  | | [7]^b^ |
| 11d | E(b)=-185  E(Q/Q^.-^) = -160 | 2.72 | 2.4⋅10^4^ s^-1^ | pH = 7 | | [5.8]^a^  [6]^c^ |
| 12d |  | 0.204 µM^-1e^ | 10^3^ µM^-1^⋅s^-1^ | pH = 7 | | [9]^b^ |
| 18d | E(b)=-185  E(QH^.^/QH^-^) = 190 | 3.269∙10^6^ | 2.4⋅10^4^ s^-1^ |  | | [5,8]^a^  [6]^c^ |
| 19d |  | 7.24∙10^4^ μM^–1e^ | 10^3^ µM^-1^⋅s^-1^ | pH = 7 | | [9]^b^ |
| 20d |  | 0.9 µM | 10^3^ s^-1^ |  | | [7]^b^ |
| **Superoxide anion (O_2_^.-^) production by Disintegrated SDHC/SDHD subunits** | | | | | | |
| 26d | E(O_2_/O_2_^.-^) = -160  E(Q/Q^.-^) = -160 | 1 | 0.05 µM^-1^⋅s^-1^ |  | | [10]^a^  [8]^a^ |

^a^ The reference for the midpoint redox potential E_M_.

^b^ The reference for the equilibrium constant K_eq_.

^c^ The reference for the rate constant of direct reaction k_forward_.

^d^ The used value of K_eq2_ is calculated from the relation K_eq1_ ∙ K_eq2_ ∙ K_eq3_ = exp (2 ∙ F ∙ (E (FAD / FADH2) – E (fum / suc)) / R ∙ T) = 0.0018 according to the thermodynamic cycle, where midpoint redox potentials E (FAD / FADH2) = –79 mV [2] (pH 7.0) and E (fum / suc) = 0 mV [13] (pH 7.0), respectively, and F, R, and T have the usual meaning.

^e^ Equilibrium constants K_eq12_ and K_eq12d_ used here correspond to pK_a_ = 5.31 for the pair QH**^.^**^.^/ Q^.–^, taken from [9]. К_eq12_ = К_eq12d_ = 10^5.31^ М^–1^ = 0.204 μM^–1^. By analogy, equilibrium constants K_eq19_ and K_eq19d_ correspond to pK_a_ = 10.86 for the pair QH2^.^/ QH^-^ [9]. К_eq19_ = К_eq19d_ = 10^10.86^ М^–1^ = 7.24∙10^4^ μM^–1^.

^f^The used value was taken from [11], which was calculated from experimental data on Mn–SOD activity in mitochondria of cardiac cells [14].

^g^The value of K_eq30_ determines relation between CIIt, ABt and CDt in steady states and depends on many factors, for instance, such as cardiolipin, mitochondrial pH and Ca^2+^ concentration. The value K_eq30_ = 5.487⋅10-3 used in computations for mixed, assembled and disintegrated, CII (Fig.4 in the main text) results in the following stationary concentrations: CIIt=100, ABt=135 and CDt=135 μM.

*Conserved moieties* *(in µM****).*** The pool of electron carriers. According to [15], the content of complex II in cardiac mitochondria is 0.209 nmol complex II/mg of mitochondrial protein. Translation of whole membrane concentration expressed in nmol/mg mit.prot. to local protein concentration expressed in μM presented in [11]. We have shown earlier [11] that 1 nmol/mg of protein corresponds to 273 μM when normalized to the mitochondrial volume (V_mit_). If the concentration is normalized to the inner mitochondrial membrane volume (V_imb_) it should be additionally taken into account that ratio W_imb_=V_imb_/V_mit_=0.24 [11]. Therefore 0.209 nmol complex II/mg of mitochondrial protein corresponds approximately to 235 μM if it is recalculated to the concentration in the inner MM (0.209 ∙ 273 / 0.24 = 237). So, we suggested in the present study that the total concentration of all redox centers localized in complex II, that is, [FAD], [2Fe–2S], [4Fe–4S], and [3Fe–4S], equal to 235 μM. The total concentration of coenzyme Q in the inner membrane was taken to be of 4541 μM as in [11].

**Supplementary Table S3. Ajusted parameter values for the model.**

| Parameter | Values |
| --- | --- |
| K_eq1_ | 0.61 µM^-1^ |
| K_eq2_* | 6.7∙10^-5^ |
| k_3_ | 4∙10^3^ s^-1^ |
| K_eq3_ | 43.89 µM |
| k_10_ | 2.47 µM^-1^⋅s^-1^ |
| K_eq10_ | 16.61 µM^-1^ |
| k_22_ | 0.027 µM^-1^⋅s^-1^ |
| k_23_ | 0.019 µM^-1^⋅s^-1^ |
| k_24_ | 0.2 µM^-1^⋅s^-1^ |
| k_25_ | 10^-4^ µM^-1^⋅s^-1^ |
| k_26_ | 10^-5^ µM^-1^⋅s^-1^ |
| K_eq27_ | 2⋅10^5^ µM^-1^ |

***** Since the value of K_eq2_ is calculated from the relation K_eq1_ ∙ K_eq2_ ∙ K_eq3_ = exp (2 ∙ F ∙ (E (FAD / FADH2) – E (fum / suc)) / R ∙ T) = 0.0018, adjusted changes in K_eq1_ and K_eq3_ result in a change in K_eq2_.

Additionally, it should point that, although the developed model describes well simultaneously various experimental data from different, bovine [16] and rat [10] heart mitochondria with the same ajustable parameter values presented in Table S3, the total concentration of CII, CIIt, has to be different in both cases and equal to 235 and 97 μM for bovine and rat heart mitochondria, respectively.

**References**

[1] A.B. Kotlyar, A.D. Vinogradov, Interaction of the membrane-bound succinate dehydrogenase with substrate and competitive inhibitors. [Biochim. Biophys. Acta](https://www.ncbi.nlm.nih.gov/pubmed/6691982) 784 (1) (1984) 24–34.

[2] T. [Ohnishi](https://www.ncbi.nlm.nih.gov/pubmed/?term=Ohnishi%20T%5BAuthor%5D&cauthor=true&cauthor_uid=6263883), T.E. [King](https://www.ncbi.nlm.nih.gov/pubmed/?term=King%20TE%5BAuthor%5D&cauthor=true&cauthor_uid=6263883), J.C. [Salerno](https://www.ncbi.nlm.nih.gov/pubmed/?term=Salerno%20JC%5BAuthor%5D&cauthor=true&cauthor_uid=6263883), H. [Blum](https://www.ncbi.nlm.nih.gov/pubmed/?term=Blum%20H%5BAuthor%5D&cauthor=true&cauthor_uid=6263883), J.R. [Bowyer](https://www.ncbi.nlm.nih.gov/pubmed/?term=Bowyer%20JR%5BAuthor%5D&cauthor=true&cauthor_uid=6263883), T. [Maida,](https://www.ncbi.nlm.nih.gov/pubmed/?term=Maida%20T%5BAuthor%5D&cauthor=true&cauthor_uid=6263883) Thermodynamic and electron paramagnetic resonance characterization of flavin in succinate dehydrogenase. [J. Biol. Chem.](https://www.ncbi.nlm.nih.gov/pubmed/6263883)  256 (11) (1981) 5577–5582.

[3] T. Ohnishi, J.C. Salerno,[Thermodynamic and EPR characteristics of two ferredoxin-type iron–sulfur centers in the succinate–ubiquinone reductase segment of the respiratory chain,](https://www.ncbi.nlm.nih.gov/pubmed/178655) J. Biol. Chem. 251 (7) (1976) 2094–2104.

[4] T. [Ohnishi](https://www.ncbi.nlm.nih.gov/pubmed/?term=Ohnishi%20T%5BAuthor%5D&cauthor=true&cauthor_uid=178656), J. [Lim](https://www.ncbi.nlm.nih.gov/pubmed/?term=Lim%20J%5BAuthor%5D&cauthor=true&cauthor_uid=178656), D.B. [Winter,](https://www.ncbi.nlm.nih.gov/pubmed/?term=Winter%20DB%5BAuthor%5D&cauthor=true&cauthor_uid=178656) T.E. [King,](https://www.ncbi.nlm.nih.gov/pubmed/?term=King%20TE%5BAuthor%5D&cauthor=true&cauthor_uid=178656) Thermodynamic and EPR characteristics of a HiPIP-type iron–sulfur center in the succinate dehydrogenase of the respiratory chain, [J. Biol. Chem.](https://www.ncbi.nlm.nih.gov/pubmed/?term=ohnishi+AND+lim+%5Bau%5D+AND+1976) 251 (7) (1976) 2105–2109.

[5] L. Yu, J. X. Xu, P.E. Haley, C.A. Yu, Properties of bovine heart mitochondrial cytochrome

b560, J. Biol. Chem. 262 (1987) 1137−1143.

[6] R.F. Anderson, S.S. Shinde, R. Hille, R.A. Rothery, J.H. Weiner, S. Rajagukguk,

E. Maklashina, G. Cecchini, [Electron-transfer pathways in the heme and quinone-binding domain of complex II (succinate dehydrogenase)](https://www.ncbi.nlm.nih.gov/pubmed/24559074), Biochemistry 53 (10) (2014) 1637–1646.

[7] C. [Hägerhäll,](https://www.ncbi.nlm.nih.gov/pubmed/?term=H%C3%A4gerh%C3%A4ll%20C%5BAuthor%5D&cauthor=true&cauthor_uid=9210286) Succinate:quinone oxidoreductases. Variations on a conserved theme.

[Biochim. Biophys. Acta](https://www.ncbi.nlm.nih.gov/pubmed/?term=hagerhall+c+%5Bau%5D+AND+1997) 1320 (2) (1997) 107–141.

[8] P.R. [Rich,](https://www.ncbi.nlm.nih.gov/pubmed/?term=Rich%20PR%5BAuthor%5D&cauthor=true&cauthor_uid=6322844) Electron and proton transfers through quinones and cytochrome *bc* complexes, [Biochim. Biophys. Act](https://www.ncbi.nlm.nih.gov/pubmed/?term=rich+pr+%5Bau%5D+AND+1984)a 768 (1) (1984) 53–79.

[9] R. Hasegawa, K . Saito, T. Takaoka, H. Ishikita, pK_a_ of ubiquinone, menaquinone, phylloquinone, plastoquinone, and rhodoquinone in aqueous solution, [Photosynth. Res.](https://www.ncbi.nlm.nih.gov/pubmed/28831654) 133(1-3) (2017) 297-304. doi: 10.1007/s11120-017-0382-y.

[10] M.P. Murphy, [How mitochondria produce reactive oxygen species](https://www.ncbi.nlm.nih.gov/pubmed/19061483), Biochem. J. 417 (1)

(2009)1–13.

[11] N.I [Markevich](https://www.ncbi.nlm.nih.gov/pubmed/?term=Markevich%20NI%5BAuthor%5D&cauthor=true&cauthor_uid=25868872), J.B. [Hoek](https://www.ncbi.nlm.nih.gov/pubmed/?term=Hoek%20JB%5BAuthor%5D&cauthor=true&cauthor_uid=25868872), Computational modeling analysis of mitochondrial superoxide production under varying substrate conditions and upon inhibition of different segments of the electron transport chain, [Biochim. Biophys. Acta](https://www.ncbi.nlm.nih.gov/pubmed/?term=markevich+ni+%5Bau%5D+AND+2015) 1847 (6–7) (2015) 656–679.

[12] N.I. Markevich, J.B. Hoek, Computational modeling analysis of acute and chronic ethanol-

induced oxidative stress, Math. Biol. Bioinformat. 9 (1) (2014) 63–88.

[13] V.G. Grivennikova, V.S. Kozlovsky, A.D. Vinogradov, Respiratory complex II: ROS

production and the kinetics of ubiquinone reduction, [Biochim. Biophys. Acta](https://www.ncbi.nlm.nih.gov/pubmed/?term=Grivennikova+AND+Kozlovsky+AND+Vinogradov+%5Bau%5D+AND+2016) 1858 (2)

(2017) 109–117.

[14] Z.Q. [Jin](https://www.ncbi.nlm.nih.gov/pubmed/?term=Jin%20ZQ%5BAuthor%5D&cauthor=true&cauthor_uid=15681709), [Zhou H.Z](https://www.ncbi.nlm.nih.gov/pubmed/?term=Zhou%20HZ%5BAuthor%5D&cauthor=true&cauthor_uid=15681709)., G. [Cecchini](https://www.ncbi.nlm.nih.gov/pubmed/?term=Cecchini%20G%5BAuthor%5D&cauthor=true&cauthor_uid=15681709), M.O. [Gray](https://www.ncbi.nlm.nih.gov/pubmed/?term=Gray%20MO%5BAuthor%5D&cauthor=true&cauthor_uid=15681709), J.S. [Karliner](https://www.ncbi.nlm.nih.gov/pubmed/?term=Karliner%20JS%5BAuthor%5D&cauthor=true&cauthor_uid=15681709), MnSOD in mouse heart:

Acute responses ischemic preconditioning and ischemia–reperfusion injury, [Am. J. Physiol. Heart. Circ. Physiol.](https://www.ncbi.nlm.nih.gov/pubmed/?term=jin+zq+AND+zhou+hz+%5Bau%5D+AND+2005) 288 (6) (2005) H2986–2994.

[15] A.P. Wojtovich, K.W. Nehrke, P.S. Brookes, [The mitochondrial complex II and ATP-sensitive potassium channel interaction: Quantitation of the channel in heart mitochondria,](https://www.ncbi.nlm.nih.gov/pubmed/21103454) Acta Biochim. Pol. 57 (4) (2010) 431–434.

[16] I. Siebels, S. [Dröse,](https://www.ncbi.nlm.nih.gov/pubmed/?term=Dr%C3%B6se%20S%5BAuthor%5D&cauthor=true&cauthor_uid=23800966) Q-site inhibitor induced ROS production of mitochondrial complex II is attenuated by TCA cycle dicarboxylates. Biochim. Biophys. Acta. 1827 (2013) 1156-1164.

**Supplementary Figures**


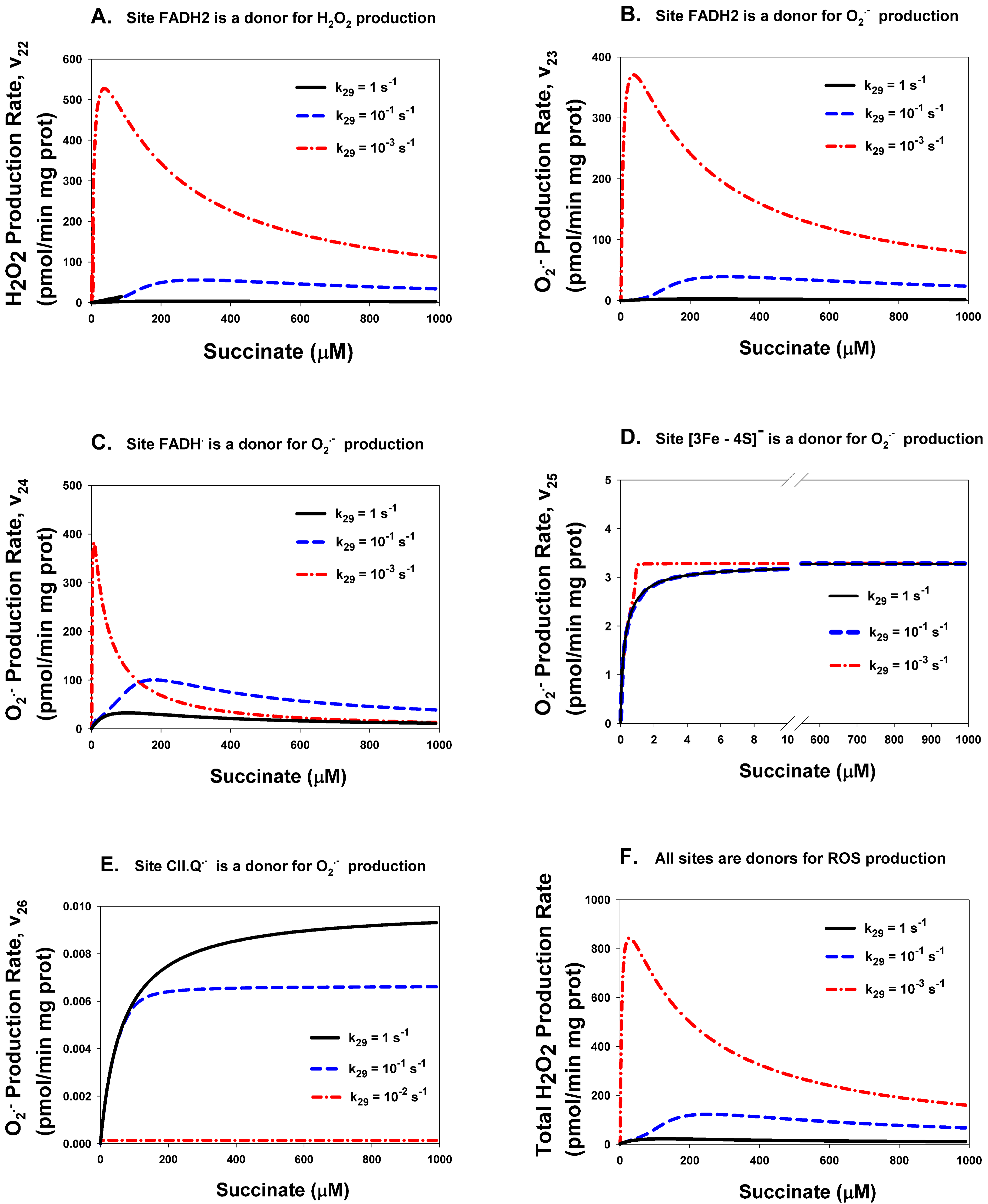


**Supplementary Figure S1. Computer simulation of the effect of stigmatellin on the kinetics of ROS production by different sites of the assembled Complex II.** The effect of stigmatellin was simulated by a decrease in the rate constant k_29_ oxidation of QH_2_ onto Q in the inner membrane. Values of k_29_ are shown at the Figure S1. Black solid curves correspond to the k_29_=1 s^-1^, blue dashed curves – k_29_=10^-1^ s^-1^, red dash-dot curves – k_29_=10^-3^ s^-1^. The total concentration of CII, CIIt, is 97 μM. The rest model parameters are presented in Supplementary Tables S2 and S3.


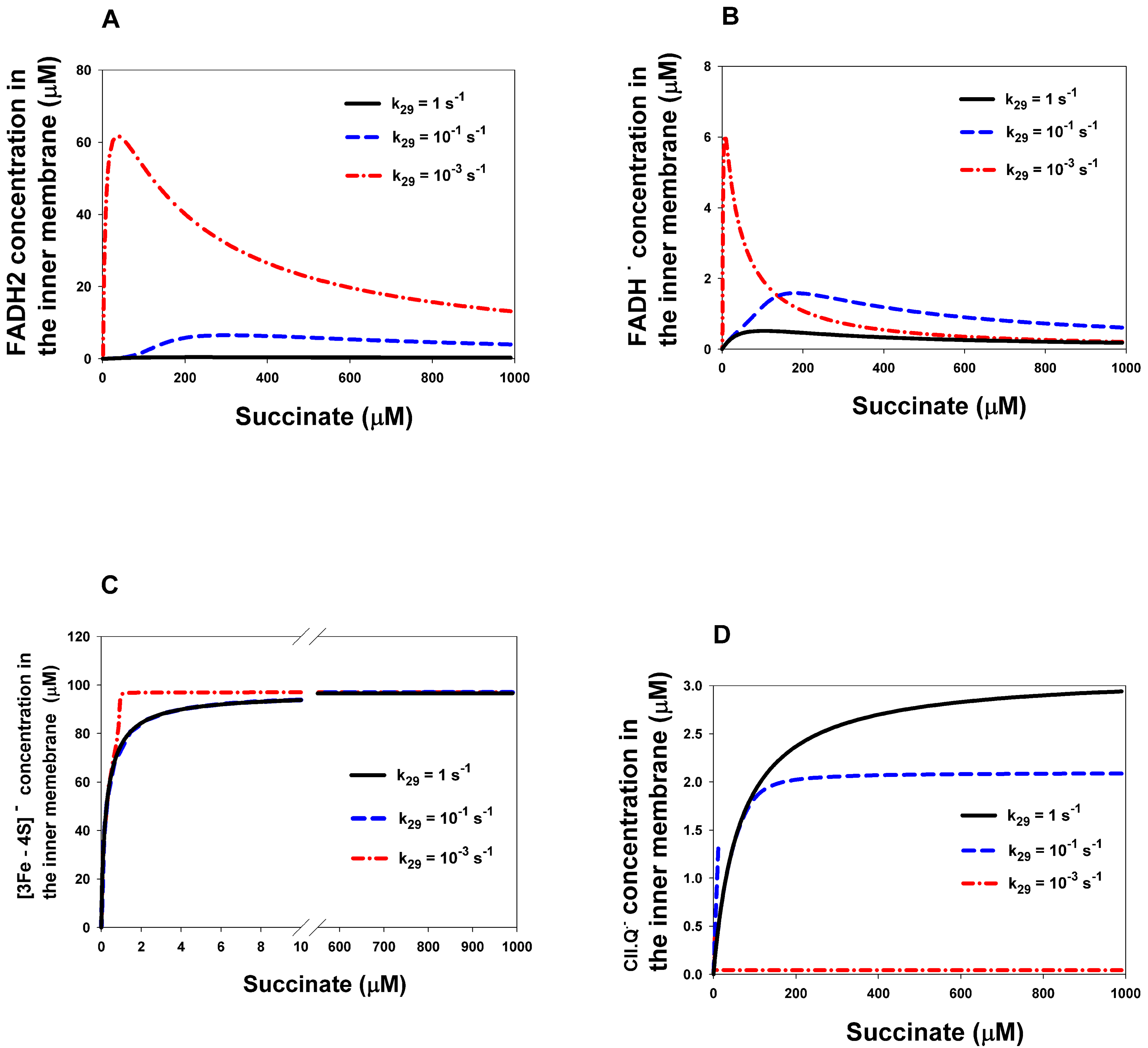


**Supplementary Fig. S2. Computer simulation of the effect of stigmatellin on the concentration of different ROS producing sites of the assembled Complex II.** The effect of stigmatellin was also as in Figure S1 simulated by a decrease in the rate constant k_29_ oxidation of QH_2_ onto Q in the inner membrane. Values of k_29_ are shown at the Figure S2. Black solid curves correspond to the k_29_=1 s^-1^, blue dashed curves – k_29_=10^-1^ s^-1^, red dash-dot curves – k_29_=10^-3^ s^-1^. All parameter values are the same as in Fig. S1.


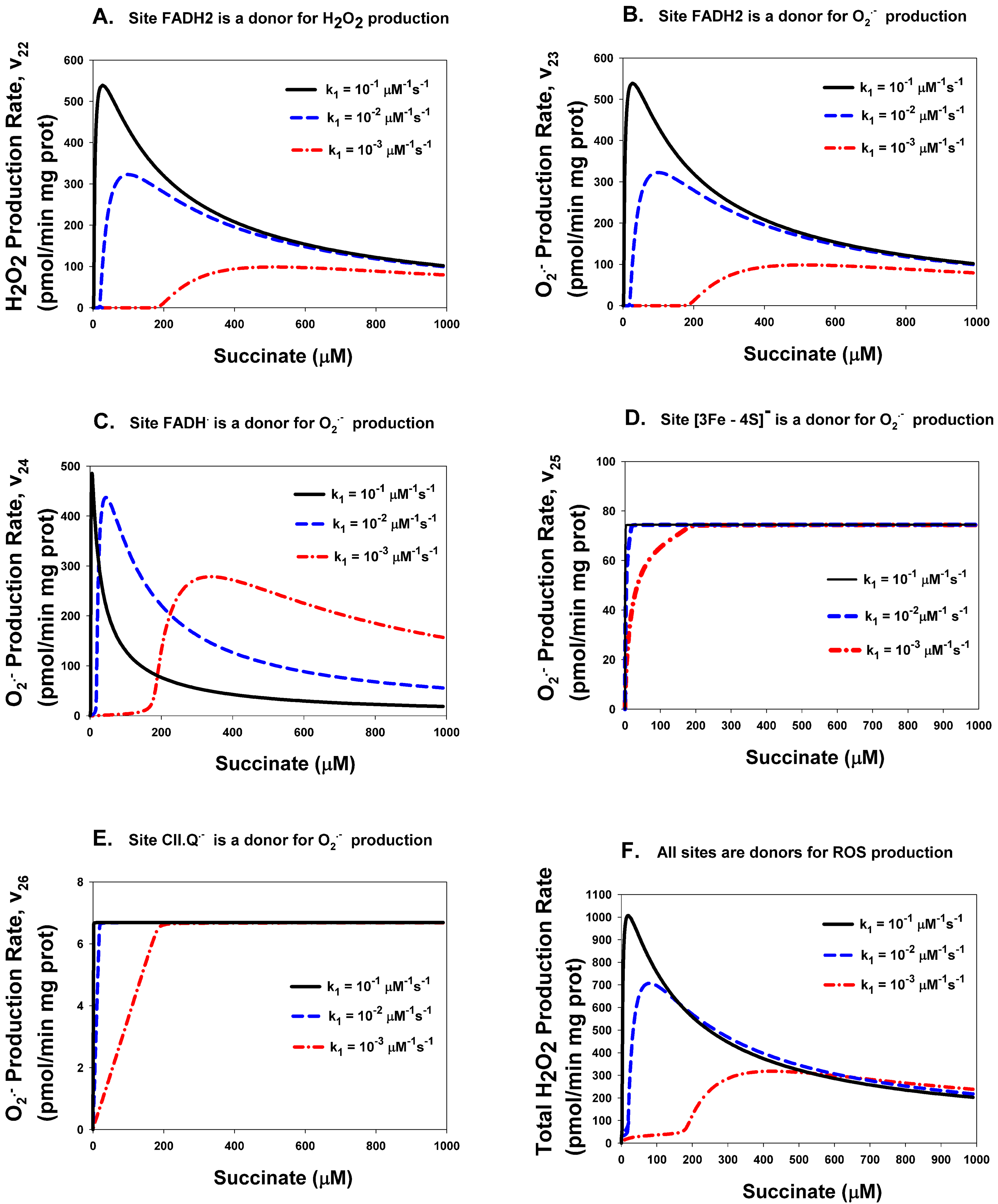


**Supplementary Figure S3. Computer simulation of the effect of changes in the rate constant k_1_ binding of succinate to FAD on the kinetics of ROS production by different sites of the assembled Complex II.** Values of k_1_ are shown at the Figure S3. Black solid curves correspond to the k_1_=10^-1^ s^-1^, blue dashed curves – k_1_=10^-2^ s^-1^, red dash-dot curves – k_1_=10^-3^ s^-1^. The rest model parameters are presented in Supplementary Table S2.


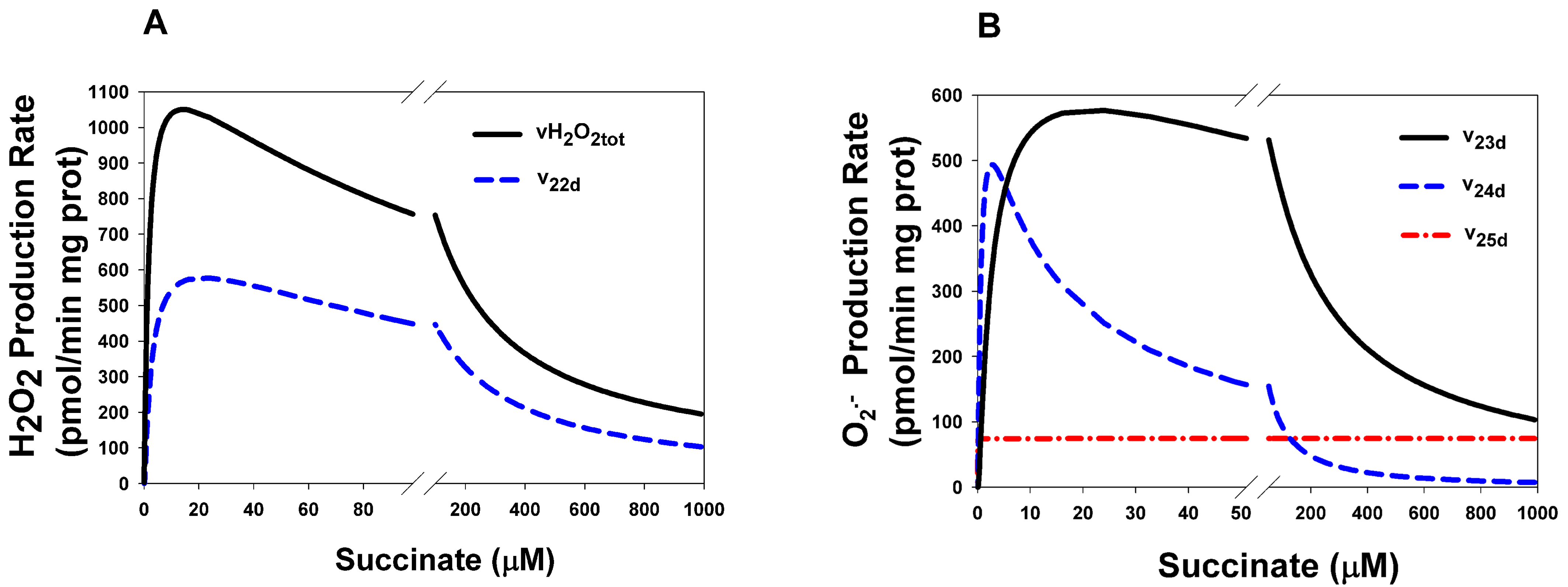


**Supplementary Figure S4. Computer simulated stationary rates of O_2_^.-^ and H_2_O_2_ production by different sites in dissociated CDHA/CDHB subcomplex of completely disintegrated CII. (A)** The rate of H_2_O_2_ production by FADH_2_ (v_22d_) and the total rate of H_2_O_2_ production by all sites (vH_2_O_2tot_) in CDHA/CDHB subcomplex of completely disintegrated CII; **(B)** The rates of O_2_^.-^ production by the following sites in CDHA/CDHB subcomplex of completely disintegrated CII: FADH_2_ (v_23d_); FADH^∙^(v_24d_); reduced [3Fe-4S]^-^ cluster (v_25d_).

Completely disintegrated CII means that all CDHA/CDHB subcomplexes of CII are dissociated from the membrane and located only in the matrix with the concentration of 235 µM.

The black solid curve in Figure S4A corresponds to the total rate of H_2_O_2_ production by CII (vH_2_O_2tot_) that was computed as the rate of H_2_O_2_ release from the mitochondrial matrix to cytosol that equal to the summary rate of H_2_O_2_ production by FADH_2_, v_22d_, and dismutation of O_2_^.-^ , v_27_, in the matrix at the steady state (see below *Explicit functions* in Mathematical model in the present Supplementary Data). The blue dashed curve in Figure S4A corresponds to the rate of H_2_O_2_ production by FADH_2_, v_22d_.

The black solid curve in Figure S4B corresponds to the v_23d_, the blue dashed curve - v_24d_, the red dash-dot curve - v_25d_.

All model parameters are presented in Supplementary Table S2.

**Mathematical model.**

A computational model corresponding to the kinetic schemes in Figure 1 in the main text and Supplementary Tables S1 and S2 consists of 35 ordinary differential equations (ODE) and 15 moiety conservation equations. The model was implemented in DBSolve Optimum software available at website [**http://insysbio.ru**](http://insysbio.ru)**.**

Additionally, the model is presented in SBML format by separate file: **2019_cII_Final.xml** as Supporting information. It should point out that order of expressions for dependent variables after recover the model from SBML format has to be exactly as in this Supplementary Data. See below *Expressions for dependent variables.*

*The system of ODE.* The system of ODE that was analized computationally in the present study can be written as follows:

d(FAD)/dt =-V_1_-V_4_+V_14_+V_22_+V_24_;

d(FAD_suc)/dt =V_1_-V_2_;

d(FADH2_fum)/dt =V_2_-V_3_;

d(FADH2)/dt =V_3_-V_5_-V_6_-V_22_-V_23_;

d(FAD_fum)/dt =V_4_;

d(FADH2_suc)/dt =V_5_;

d(FeS2)/dt =-V_6_+V_7_-V_14_+V_15_;

d(CD_QH2)/dt =V_19d_-V_20d_;

d(CD_QH**^∙^**)/dt =V_12d_-V_18d_;

d(FeS4)/dt =-V_7_+V_8_-V_15_+V_16_;

d(CD_Q^.–^)/dt =V_11d_-V_12d_-V_26d_;

d(FeS3)/dt =-V_8_+V_9_+V_13_-V_16_+V_17_+V_21_+V_25_;

d(b_d)/dt =V_11d_+V_18d_;

d(b)/dt =-V_9_+V_11_-V_17_+V_18_;

d(CD_Q)/dt =V_10d_-V_11d_+V_26d_;

d(Q)/dt =-V_10_+V_29_-V_10d_;

d(CII)/dt =-V_10_+V_20_ - V_27_;

d(CII_AA5)/dt = V_27_

d(CII_Q)/dt =V_10_-V_11_-V_13_+V_26_;

d(CII_Q^.–^)/dt =V_11_-V_12_+V_13_-V_26_;

d(CII_QH**^∙^**)/dt =V_12_-V_18_-V_21_;

d(CII_QH)/dt =V_18_-V_19_+V_21_;

d(FeS3m_d)/dt =V_8d_+V_16d_-V_25d_;

d(QH2)/dt =V_20_-V_29_+V_20d_;

d(H_2_O_2_)/dt = Wimb/Wmx * (V_22_+V_22d_) +V_27_-V_28_;

d(Q^.–^)/dt = Wimb/Wmx * (V_23_ +V_24_ +V_25_ +V_26_ +V_23d_ + V_24d_ +V_25d_ +V_26d_) – 2*V_27_;

d(ABt)/dt =-V_30_;

d(FeS4m_d)/dt =V_7d_-V_8d_+V_15d_-V_16d_;

d(FeS2_d)/dt =-V_6d_+V_7d_-V_14d_+V_15d_;

d(FAD_d)/dt =-V_1d_-V_4d_+V_14d_+V_22d_+V_24d_;

d(FAD_suc_d)/dt =V_1d_-V_2d_;

d(FADH2_fum_d)/dt =V_2d_-V_3d_;

d(FADH2_d)/dt =V_3d_-V_5d_-V_6d_-V_22d_-V_23d_;

d(FAD_fum_d)/dt =V_4d_;

d(FADH2_suc_d)/dt =V_5d_;

The expressions for rates V_i_ (i=1, 30) in assembled CII and V_j_ (j=1d, 26d) in disintegrated CII are presented in Table S1, and all the parameters are in Table S2 of this Supplementary Data. Here, the fractional volume ratio of matrix, Vmx (Wmx=Vmx/Vmit) and inner membrane, Vimb (Wimb=Vimb/Vmit) to the total mitochondrial volume Vmit equal approximately 0.24 and 0.652, respectively [10].

*Conserved moieties* *(in µM).* The model took into account the laws of conservation of the total concentration in the membrane of both complex II and the pools of different redox centers.

The total concentration of ubiquinone binding centers, CD, in SDHC/SDHD complex of disintegrated CII at different states are:

Pool[1] = CD + CD_QH2 + CD_ H**^∙^** + CD_ Q^∙–^ + CD_Q + CD_QH^─^ = CDt;

The total concentration of the ubiquinone pool at different states:

Pool[2] = QH2 + Q + CII_Q + CII_Q^∙–^ + CII_QH^∙^ + CII_QH^─^ + CII_QH2 + CD_Q + CD_Q^∙–^ + CD_QH^∙^ + CD_QH^─^ + CD_QH2 = Qtot;

The total concentration of cyt *b* in SDHC/SDHD complex of disintegrated CII at different states is:

Pool[3] = b^–^_d + b_d =CDt;

Total concentration of FAD centers in SDHA/SDHB complex of disintegrated CII at different states is:

Pool [4] = FADHd**^∙^** + FADd + FADd_suc + FADH2d_fum + FADH2d + FADd_fum + FADH2d_suc =ABt;

The total concentration of [3Fe–4S] clusters at different oxidized and reduced states in SDHA/SDHB complex of disintegrated CII:

Pool [5] = [3Fe–4S]d^–^ + [3Fe–4S]d = ABt;

The total concentration of [4Fe–4S] clusters at different oxidized and reduced states in SDHA/SDHB complex of disintegrated CII:

Pool[6] = [4Fe–4S]d^–^ + [4Fe–4S]d = ABt;

The total concentration of [2Fe–2S] clusters at different oxidized and reduced states in SDHA/SDHB complex of disintegrated CII:

Pool[7] = [2Fe–2S]d^–^ + [2Fe–2S]d = ABt;

Total concentration of SDHC/SDHD complexes in assembled and disintegrated CII:

Pool[8] = CDt + CIIt;

The total concentration of SDHA/SDHB complexes in assembled and disintegrated CII:

Pool[9] = CIIt + ABt;

Total concentration of ubiquinone binding centers, CII, in assembled CII at different states is:

Pool[10] = CII_QH2 + CII + CII_AA5 + CII_Q + CII_Q^∙–^ + CII_QH**^∙^** + CII_QH^─^ = CIIt;

The total concentration of cyt *b* in assembled CII at different states is:

Pool[11] = b^–^ + b = CIIt;

The total concentration of [3Fe–4S] clusters at different oxidized and reduced states in assembled CII:

Pool[12] = [3Fe–4S]^–^ + [3Fe–4S] = CIIt;

The total concentration of [4Fe–4S] clusters at different oxidized and reduced states in assembled CII:

Pool[13] = [4Fe–4S]^–^ + [4Fe–4S] = CIIt;

The total concentration of [2Fe–2S] clusters at different oxidized and reduced states in assembled CII:

Pool[14] = [2Fe–2S]^–^ + [2Fe–2S] = CIIt;

The total concentration of FAD centers at different states in assembled CII is:

Pool[15] = FADH**^∙^** + FAD + FAD_suc + FADH2_fum + FADH2 + FAD_fum + FADH2_suc = CIIt;

The concentration of pools of all redox centers in assembled and disintegrated CII is taken to be equal to the concentration of corresponding SDHA/SDHB and SDHC/SDHD complexes, i.e. 1 redox center per 1 SDHA/SDHB or SDHC/SDHD complex.

So, total concentration of assembled and disintegrated CII: Pool[8]=Pool[9]= 235 µM.

Pool[2] = 4541 µM; Pool[1] = Pool[3] = CDt; Pool[4] = Pool[5] = Pool[6] = Pool[7] = ABt;

Pool[10] = Pool[11] = Pool[12] = Pool[13] = Pool[14] ] = Pool[15] = CIIt, where the total stationary concentrations CIIt, ABt and CDt are taken 100, 135 and 135 µM, respectively, that corresponds to K_eq30_=5.487∙10^-3^ µM^-1^.

The concentration of AA5, inhibitor of the Q-binding site, is taken unchanged in time, i.e. it is used in the model as a parameter.

*Expressions for dependent variables.* The expressions for the concentration of all 15 dependent variables used in ODE are easily calculated from the pools of different variables and are presented below.

CD_QH = - CD_QH2 – CD_QH**^∙^** - CD_Q^∙–^ - CD_Q – Q – CII_Q – CII_Q^∙–^ - CII_QH**^∙^** - CII_QH^─^ - QH2 – CII_QH2 + Pool[2];

CD = - CD_QH2 – CD_QH**^∙^** - CD_Q^∙–^ - CD_Q – CD_QH^─^ + Pool[1];

b^─^_d = - b_d + Pool[3];

FADHd**^∙^** = - FADd – FADd_suc – FADH2d_fum – FADH2d – FADd_fum –FADH2d_suc + Pool[4];

[3Fe–4S]d = - [3Fe–4S]d^─^ + Pool[5];

[Fe–4S]d = - [4Fe–4S]d^─^ + Pool[6];

[2Fe–2S]d^─^ = - [2Fe–2S]d + Pool[7];

CIIt = - ABt + Pool[9];

CDt = - CIIt + Pool[8];

CII_QH2 = - CII – CII_AA5 – CII_Q – CII_Q^∙–^ - CII_QH**^∙^** - CII_QH^─^ + Pool[10];

b^─^ = - b + Pool[11];

[3Fe–4S]^─^ = - [3Fe–4S] + Pool[12];

[4Fe–4S]^─^ = - [4Fe–4S] + Pool[13];

[2Fe–2S]^─^ = - [2Fe–2S] + Pool[14];

FADH^─^ = - FAD – FAD_suc – FADH2_fum – FADH2 – FAD_fum – FADH2_suc + Pool[15];

*Explicit functions.* Expressions for some important functions used in computations are presented below.

VO_2_^∙–^_tot_ = V_23_ + V_24_ + V_25_ + V_26_ + V_23d_ + V_24d_ + V_25d_ + V_26d_; the total rate of O_2_^∙–^ production by assembled and disintegrated CII in µM/s.

VH_2_O_2tot_ = (V_22_ + V_22d_)*Wimb/Wmx + V_28_; the total rate of H_2_O_2_ production by assembled and disintegrated CII in µM/s expressed as WHOLE MITO Rates (Wimb=Vimb/Vmit, where Vimb and Vmit are volumes of the inner membrane and whole mitochondria, respectively) and Wimb=0.24 [10].

vO_2_^∙–^_tot_ = VO_2_^∙–^_tot_ *220*Wimb; The total rate of O_2_^∙–^ production normalized to the total mitochondria volume in order to compare with experimental data, in pmole/min/mg prot, 1 μM/s = 220 pmol/min mg mitochondrial protein [10].

vH_2_O_2tot_ = VH_2_O_2tot_ * 220 * Wmx; The total rate of H_2_O_2_ production normalized to the total mitochondria volume in order to compare with experimental data, in pmole/min/mg prot.

v_20_ = V_20_*220*Wimb/1000; the rate of QH_2_ production in nmole/min/mg prot

v_6_=V_6_*220*Wimb/1000; in nmole/min/mg prot.

v_6f_=V_6f_*220*Wimb/1000, where V_6f_ is the forward rate of V_6_: V_6f_ = k_6_ ∙ FADH2 ∙ [2Fe-2S];

v_6r_=V_6r_*220*Wimb/1000, where V_6r_ is the reverse rate of V_6_: V_6r_ = k_6_ ∙ FADH^∙^ ∙ [2Fe-2S]^-^ ∙ H^+^ / K_eq6_;

v_8_=V_8_*220*Wimb/1000; v_9_=V_9_*220*Wimb/1000; v_11_=V_11_*220*Wimb/1000; v_13_=V_13_*220*Wimb/1000;

Other rates expressed in nmole/min/mg prot in order to compare with experimental data.

v_22_=V_22_*220*0.24; v_23_=V_23_*220*0.24; v_23f_=V_23f_*220*0.24, where V_23f_ = k_23_ ∙ FADH2 ∙ O_2_;

v_23r_=V_23r_*220*0.24, where V_23r_ = k_23_ ∙FADH^∙^ ∙ O_2_**^.^**^-^ ∙ H^+^ / K_eq23_;

v_24_=V_24_*220*0.24; v_25_=V_25_*220*0.24; v_26_=V_26_*220*0.24;

v_22d_=V_22d_*220*0.24; v_23d_=V_23d_*220*0.24; v_24d_=V_24d_*220*0.24; v_25d_=V_25d_*220*0.24;

v_26d_=V_26d_*220*0.24.
